# Information on disease resistance patterns of grape varieties may improve disease management

**DOI:** 10.3389/fpls.2022.1017658

**Published:** 2022-11-14

**Authors:** Irene Salotti, Federica Bove, Tao Ji, Vittorio Rossi

**Affiliations:** ^1^ Department of Sustainable Crop Production (DI.PRO.VES.), Università Cattolica del Sacro Cuore, Piacenza, Italy; ^2^ Horta Srl, Piacenza, Italy

**Keywords:** plasmopara viticola, erysiphe necator, phyllosticta ampelicida, disease management, vitis vinifera

## Abstract

Resistance to downy mildew (DM) and powdery mildew (PM) contributes to sustainable vineyard management by reducing the diseases and the need for fungicide applications. Resistant varieties vary in their degree of resistance to DM and PM, and in their susceptibility to other diseases. As a consequence, fungicide use may differ among varieties depending on their “resistance patterns” (i.e., the resistance level of a variety toward all of the diseases in the vineyard). The resistance patterns of 16 grapevine varieties to DM, PM, black rot (BR), and gray mold (GM) were evaluated over a 4-year period under field conditions. Disease severity was assessed on leaves and bunches, and the AUDPC (Area Under Disease Progress Curve) was calculated to represent the epidemic progress. GM was found only on bunches and only at very low levels, irrespective of the year or variety, and was therefore excluded from further analyses. The varieties were then grouped into four resistance patterns: i) low resistance to DM and PM, intermediate resistance to BR; ii) high resistance to DM, intermediate resistance to PM, low resistance to BR; iii) intermediate resistance to DM and BR, low resistance to PM; and iv) high resistance to DM, PM, and BR. AUDPC values on leaves were positively correlated with AUDPC values on bunches for susceptible varieties but not for resistant ones, with the exception of PM. Therefore, bioassays with leaves can be used to predict the resistance of bunches to DM and BR for susceptible varieties but not for resistant ones. These results may facilitate both strategic and tactical decisions for the sustainable management of grapevine diseases.

## Introduction

Downy mildew (DM, caused by *Plasmopara viticola* (Berk. & M.A. Curtis) Berl. & De Toni), powdery mildew (PM, *Erysiphe necator* (Schwein.) Burrill), grey mold (GM, *Botrytis cinerea* Pers.), and black-rot (BR, *Phyllosticta ampelicida* (Engleman) Van der Aa) are the main foliar and bunches diseases affecting grape production worldwide. Under favourable weather conditions, they cause substantial yield and quality losses (Scribner and Viala, 1888; Ferrin and Ramsdell, 1978; Pallotta et al., 1998; Calonnec et al., 2004; Hoffman et al., 2004; Jermini et al., 2010).

Disease control in vineyards is generally achieved by using fungicides, copper, and sulphur, all of which have negative effects on human health, the vineyard environment, and biodiversity (Rusch et al., 2015; La Torre et al., 2018; Lamichhane et al., 2018; Reiff et al., 2021). As a consequence, safer disease control methods are needed. The use of mathematical models and Decision Support Systems (DSSs) to improve the scheduling of plant protection product application, as well as the use of biocontrol agents, botanicals, and natural resistance inducers (Cohen et al., 1999; Hamiduzzaman et al., 2005; Caffi et al., 2007; Rossi et al., 2014; Pertot et al., 2017; Rossi et al., 2019) are sustainable alternatives that can be combined for effective Integrated Pest Management (IPM) in vineyards. Resistant grapevine varieties are also a promising component of IPM (Lu, 1997). Since the early 2000s, resistance to grape diseases has been a major concern of breeding programs (Delame et al., 2019; Miclot et al., 2019; Zini et al., 2019), and a number of resistant varieties have been developed through hybridization of *Vitis vinifera* with wild American and Asian *Vitis* species and have also been identified among varieties of *V. vinifera* from Central Asia (Gessler et al., 2011; Foria et al., 2019). At present, more than 30 quantitative trait loci (QTL) associated with resistance to downy and powdery mildews, as well as to other harmful organisms (*Phyllosticta ampelicida*, *Diaporthe ampelina, Agrobacterium* spp., Pierce’s disease, *Daktulosphaira vitifoliae*, and *Xiphinema index*) have been identified (www.vivc.de accessed on 19 May 2022) and used in breeding programs (Di Gaspero and Cattonaro, 2010), with emphasis on DM and PM (Zini et al., 2015; Guimier et al., 2019; Schneider et al., 2019). Partial resistance to DM and PM modifies some resistance components such as infection frequency, latency period, lesion size, spore production, infectious period, and infectivity (Bove and Rossi, 2020). Therefore, partial resistance does not stop infections but reduces disease progress in the vineyard (Bove et al., 2021).

The development of resistant varieties requires screening in both the laboratory and the field. Although leaf disc bioassays have been developed for screening resistant genotypes under laboratory conditions (Vezzulli et al., 2018; Eisenmann et al., 2019; Bove and Rossi, 2020; Possamai et al., 2020), less research has been focused on the assessment of resistance on leaves and especially on bunches under field conditions. As a consequence, the information on the resistance level of bunches is quite limited.

Varieties expressing partial resistance to DM and PM require some disease control *via* fungicide application (Zambon et al., 2019) in order to avoid loss and to maintain the pathogen at low population densities so as to reduce the development of resistance-breaking genotypes (Schwander et al., 2012; Foria et al., 2019). In addition, some varieties with resistance to DM and PM are highly susceptible to other diseases like GM and BR (Foria et al., 2019), which may need control interventions. However, when a vineyard manager decides that DM- and PM-resistant varieties require fungicide applications, the manager has no or few tools for determining the intensity and the scheduling of these applications.

In this work, we characterised the “resistance patterns” of 16 grape varieties in an experimental vineyard with respect to the four main pathogens of grape: *P. viticola, E. necator, B. cinerea*, and *P. ampelicida*. Some of these varieties carry one or more Rpv (acronym for resistance to *Plasmopara viticola*) loci and/or Ren (acronym for resistance to *Erysiphe necator*) loci that confer partial resistance to DM and PM. We define the resistance pattern as the resistance level of a variety against all of the diseases (i.e., DM, PM, GM, and BR in this study) in a vineyard. The resistance pattern of a variety may affect disease management because it indicates which pathogens may develop and require light or intensive control.

## Materials and methods

### Experimental vineyard

The research was performed over four grape-growing seasons (in years 2017, 2018, 2019, and 2021) in an experimental vineyard located on the campus of Università Cattolica del Sacro Cuore in Piacenza, Northern Italy (45°02′05′′N, 9°43′46′′E). The vineyard is planted with 16 grape varieties, most of which have one or more loci conferring partial resistance to *P. viticola* (Rpv) and *E. necator* (Ren). Some of these varieties are also expected to have loci involved in the resistance to *P. ampelicida* (Rgb), but the presence of these loci has not been confirmed (Töpfer and Trapp, 2022). The *V. vinifera* variety ‘Merlot’, which is known to be highly susceptible to DM, PM, GM, and BR, served as the positive control. The 16 grapevine varieties, their pedigree, and their known resistance loci (Rpv and Ren) are listed in **Table 1**. ‘Bronner’, ‘Johanniter’, and ‘Solaris’ were developed by the Institute of Viticulture and Enology in Freiburg (Germany). The Julius Kühn Institut (JKI) in Geilweilerhof, Siebeldingen (Germany) performed the hybridization of ‘Calardis Blanc’, ‘Felicia’, ‘Villaris’, ‘Calandro’, ‘Regent’, and ‘Reberger’. ‘Merlot Khorus’, ‘Merlot Kanthus’, ‘Cabernet Volos’, and ‘Fleurtai’ varieties were developed at the University of Udine and Institute of Applied Genetics (IGA) in Italy. ‘Palava’ was developed by the OSEVA-Krajsky semenarsky podnik in Hradec Králové (Czech Republic). ‘Rkatsitelii’ is an autochthonous grapes (*V. vinifera*), cultivated in Georgia.

**Table 1:** The 16 varieties used in the study, their pedigrees, and their resistance-related loci to *Plasmopara viticola* (Rpv) and *Erysiphe necator* (Ren).

Variety	Pedigree	Loci								
		Rpv3	Rpv3^1^	Rpv3^2^	Rpv3^3^	Rpv10	Rpv12	Ren3	Ren8	Ren9
Bronner	Merzling x Geisenheim 6494				x	x		x		x
Cabernet volos	Cabernet sauvignon x 20/3						x		unknown^a^	
Calandro	Domina x Regent		x					x		x
Calardis blanc	Geilweilerhof GA-47-42 x S:V: 39-639 KL.1		x	x				x		x
Felicia	Sirius x Vidal Blanc	x						x		x
Fleurtai	Tocai x 20/3						x	x		
Johanniter	Riesling Weiss x Freiburg 589-54		x					x		x
Merlot										
Merlot Kanthus	Merlot x 20/3	x							unknown^a^	
Merlot Khorus	Merlot x 20/3						x		unknown^a^	
Palava	Traminer x Muller Thurgau									
Reberger	Regent x Lemberger							x		x
Regent	Diana x Chambourchin		x					x	x	x
Rkatsitelii	Unknown									
Solaris	Merzling x Geisenheim 6493				x	x		x		x
Villaris	Sirius x Vidal Blanc		x					x		x

a No studies are available to confirm the presence of Ren loci in the genome.

The 16 varieties were arranged in a complete randomized block design, with three plots. In each plot, there were four contiguous plants of each of the 16 varieties. The vines were 5 years old in 2017 and were managed with a single Guyot training system pruned at 10 buds; the vines were separated by 1.2 m in the row and 2.0 m between rows. In the experimental vineyard, the four diseases are usually present and cause epidemics of different severity, depending on the year. Fungicides were not applied for the entire duration of the experiment. Air temperature (T, °C), relative humidity (RH, %), rainfall (R, mm), and leaf wetness (LW, hours) were recorded by a standard meteorological station (iMetos^®^, Pessl Instruments, Austria) located in the experimental vineyard.

### Disease assessments

Diseases were assessed at 7- to 14-day intervals from stage 57 of Lorentz et al. (1995) (inflorescences fully developed, approximately mid-May) to stage 83 (berries developing colours, late August) in each season; there were 14 disease assessments in 2017 and 2021, 12 in 2018, and 8 in 2019. The incidence and severity of the four diseases were estimated on each assessment day on 33 randomly selected leaves and 33 randomly selected bunches (new leaves and bunches in each assessment) per variety per plot by using the EPPO standard diagrams (EPPO, 2000), which define classes of disease severity from 1 to 7 based on the percentage of leaf or bunch area with disease symptoms as follows: 1 = 0%; 2 = >0 to 5%; 3 = >5 to 10%; 4 = >10 to 25%; 5 = >25 to 50%; 6 = >50 to 75%; and 7 = >75 to 100%. The disease severity (DS) in each plot was finally calculated separately for leaves and bunches as indicated by Madden et al. (2007):


(1) $$DS=\;\sum_{1}^{7}\frac{\left(n_{i}\times v_{i}\right)}{N\times V}$$

where n*_i_* is the number of diseased leaves or bunches in one class; v*_i_* is the central disease severity value of the EPPO classes (2.5, 7.5, 17.5, 32.5, and 87.5% of affected leaf or bunch area); N is the total number of observed leaves or bunches; V is the central value of the highest class (i.e., 87.5%); and *i* is the subscript for the number of the class.

Disease severity data were used to calculate the area under the disease progress curve (AUDPC) on leaves and bunches by using the trapezoid method as indicated by Madden et al. (2007):


(2) $$AUDPC=\sum_{j=1}^{D_{j-1}}\frac{\left(y_{j}+y_{j+1}\right)}{2}\times (t_{j+1}-t_{j})$$

where y*_j_* + y*_j_*_+1_ is the sum of two consecutive values of disease severity; t*_j_*_+1_ – t*_j_* is the number of days between two consecutive disease assessments; D*_j_* is the total number of disease assessments during the season; and *j* is the subscript of the disease assessment.

### Data analysis

To explore the relationships between disease progress on leaves and bunches, scatter plots were drawn representing the 4-year averages and standard errors of AUDPCs on leaves (horizontal axis) and bunches (vertical axis) the plot area was then divided into quadrants based on the overall averages as follows: (i) high disease on both leaves and bunches (upper right quadrant); (ii) high disease on leaves and low disease on bunches (lower right quadrant); (iii) low disease on leaves and high disease on bunches (upper left quadrant); and (iv) low disease on both leaves and bunches (lower left quadrant).

The resistance pattern of each variety was summarized in spider graphs based on the AUDPC values for DM, PM, and BR recorded on leaves and bunches. For each disease and organ, the 4-year AUDPC values were rescaled, so that data in are expressed in a 0 to 1 scale, in which 1 is the highest AUDPC value found in the experiment.

To group grape varieties based on the resistance patterns, a multivariate hierarchical cluster analysis was performed using the AUDPC values of all of the diseases for both leaves and bunches. Clustering was conducted on data that were standardized by using the z-scores as follows: (x_i_ – x_m_)/sd, where x_i_ is any value of a variable, x_m_ is the average for the variable, and sd is the standard deviation. Clustering also involved the use of Ward’s method, in which the distance between two statistical clusters is measured by the increase in sum of squares resulting from the merging of clusters, and similarities are measured by Euclidian square difference; statistical clusters were finally identified by applying a 5-unit dissimilarity cut-off point.

To check differences among the groups of varieties found through the cluster analysis, AUDPC values were transformed using the natural logarithm function to stabilize variances and were then subjected to an analysis of variance (ANOVA). Differences indicated by significant F values were explored by the Fischer’s protected least significant difference (LSD) test at P=0.05. Data were finally back-transformed using the inverse exponential function. Relationships between the AUDPC values on leaves and bunches for each disease were finally investigated for each of the four clusters by calculating Pearson’s correlation coefficients.

All analyses were performed using IBM SPSS Statistic for Windows, version 25.0 (Armonk, NY: IBM Corp).

## Results

### Weather conditions and disease progress on the susceptible control (‘Merlot’)

**Figure 1:**
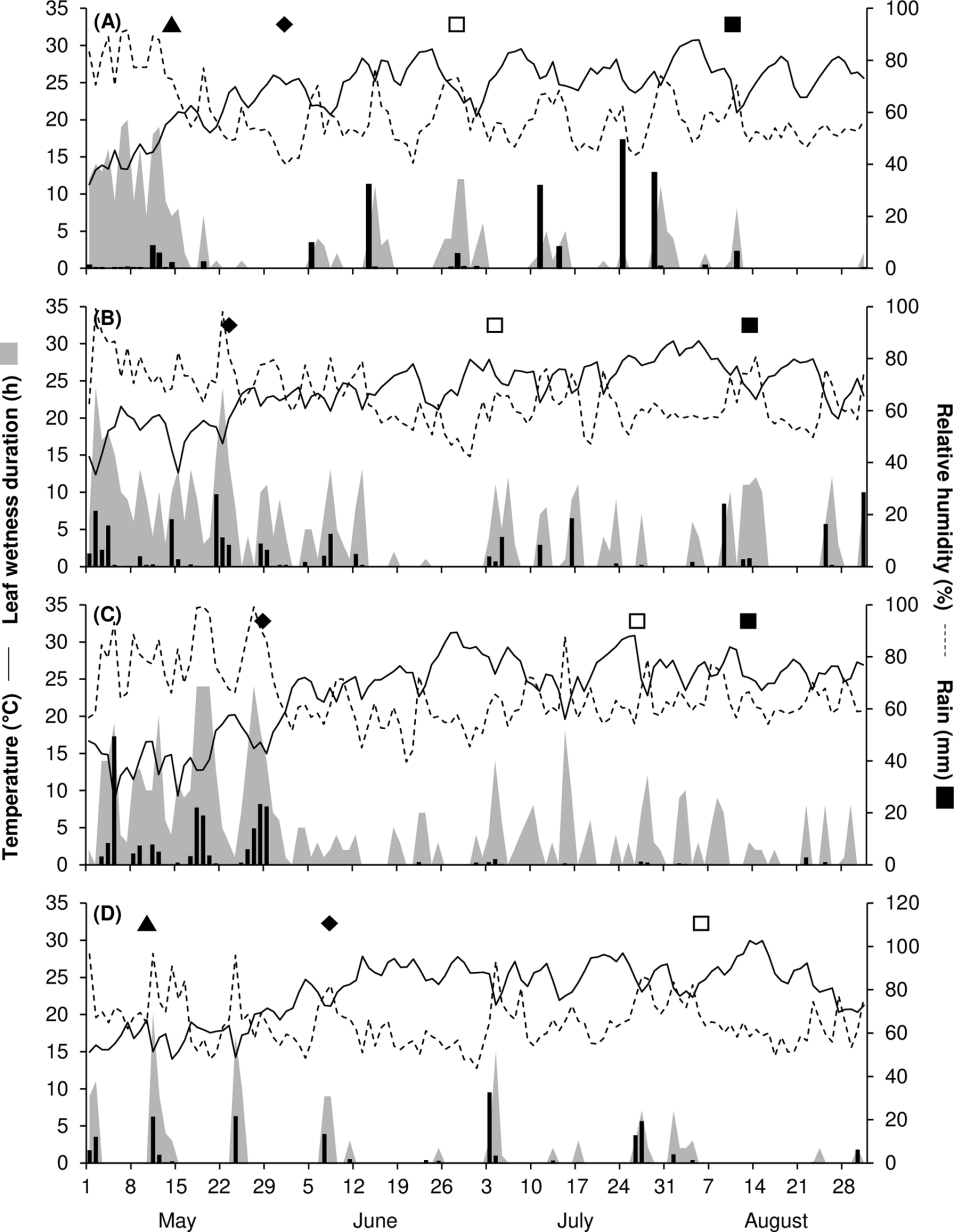
Temperature (°C, solid line), relative humidity (%, dotted line), rainfall (mm, black bars), leaf wetness (hours, grey area), and the main growth stages of vines (▲ = 5^th^ leaf unfolded – BBCH15, ♦ = full bloom – BBCH65, □ = veraison – BBCH81, ◼ = berry ripening – BBCH87) in 2017 **(A)**, 2018 **(B)**, 2019 **(C)**, and 2021 **(D)**.

**Figure 2:**
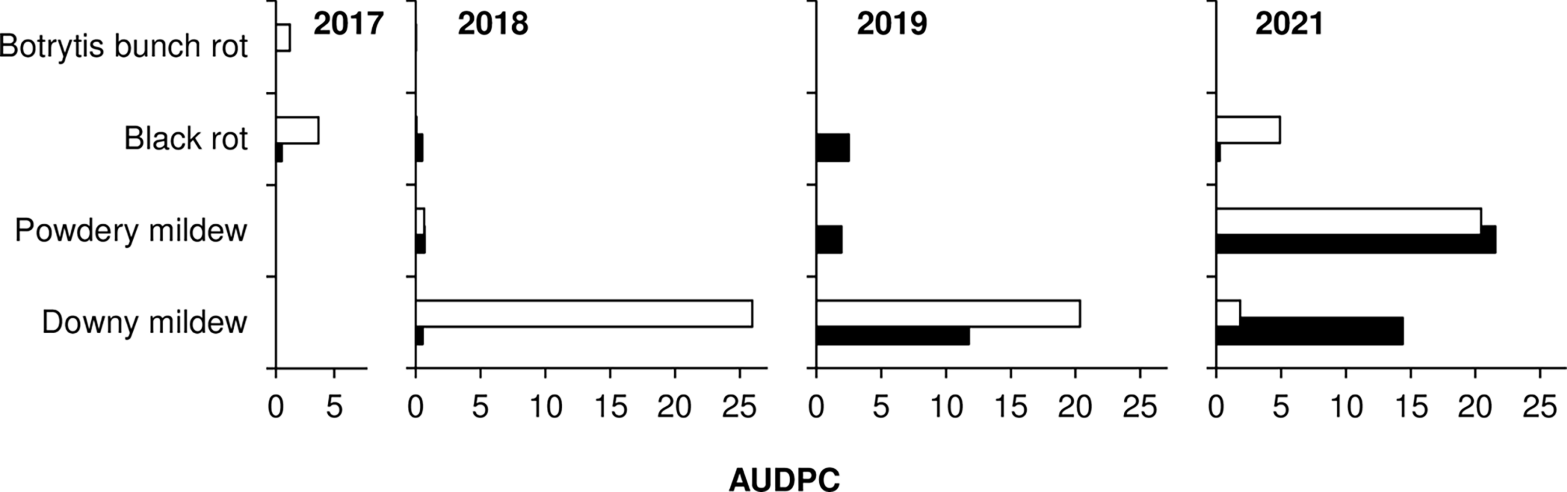
Values of AUDPC (area under the disease progress curve) on leaves (black bars) and bunches (white bars) for the susceptible variety ‘Merlot’ (the positive control) for downy mildew, powdery mildew, black-rot, and Botrytis bunch rot in 2017, 2018, 2019, and 2021.

In 2017, May to August was warm and dry, with an average temperature of 24.2°C (min = 11.3°C, max = 30.8°C), an average RH of 59%, a total of 206 mm of rain on 28 rainy days, and a total of only 341 h of leaf wetness (**Figure 1A**). On ‘Merlot’ (the susceptible control), the ‘5^th^ leaf unfolded stage’ was recorded on May 17, and full bloom occurred about 2 weeks later; veraison and berry ripening occurred on June 29 and August 10, respectively. The weather conditions in 2017 were not favourable for the development of mildews, which were present only at trace levels on the sensitive variety ‘Merlot’ (**Figure 2**). BR was then the main disease affecting grape leaves and bunches. On ‘Merlot’, the first BR symptoms were recorded at the end of May on leaves and about 1 month later on bunches; a rapid increase of the disease was observed beginning in July (**Supplementary Figure S1**), and final AUDPC values in August were 0.51 on leaves and 3.65 on bunches (**Figure 2**).

In 2018, the season was cooler and wetter than in 2017, with an average temperature of 23.8°C (min = 12.4°C, max = 30.4°C), an average RH of 65%, and a total of 562 h of leaf wetness; frequent rains occurred, with a total of 290 mm of rain on 45 days regularly distributed from May to August (**Figure 1B**). The ‘5^th^ leaf unfolded stage’ for ‘Merlot’ was recorded at the end of April, and full bloom occurred in the last third of May; veraison and berry ripening were recorded on July 3 and August 13, respectively. The weather conditions in 2018 led to the development of DM, which was first detected on ‘Merlot’ at the end of June on both leaves and bunches (**Supplementary Figure S2**). The disease increased on ‘Merlot’ during the season, with final AUDPC values of 0.53 on leaves and 25.97 on bunches (**Figure 2**). PM and BR did not develop severe epidemics, with final AUDPC values< 0.7 on both leaves and bunches of ‘Merlot’ (**Figure 2**).

The 2019 season was the coolest and wettest; the average temperature (May to August) was 23.2°C (min = 8.7°C, max = 31.3°C), with an average RH of 65%, a total of 216 mm of rain on 38 rainy days, and a total of 601 h of leaf wetness. May 2019 had frequent and intense rainfall events and prolonged periods of leaf wetness (**Figure 1C**). The ‘5^th^ leaf unfolded stage’ was observed on ‘Merlot’ at the end of April, and full bloom occurred in the last third of May; veraison and berry ripening on ‘Merlot’ were recorded on July 26 and August 28, respectively. The weather conditions in 2019 were favourable for the development of DM, which was first observed on ‘Merlot’ leaves in early June and on ‘Merlot’ bunches in late June (**Supplementary Figure S3**); a DM epidemic developed during the season, with final AUDPC values of 11.77 on leaves and 20.34 on bunches (**Figure 2**). PM was observed only on leaves, on which mild epidemics developed; at the last assessment, the AUDPC value for PM on ‘Merlot’ leaves was 1.93 (**Figure 2**). BR was detected on ‘Merlot’ leaves beginning in the first third of June (**Supplementary Figure S3**), and the disease severity then increased, resulting in a final AUDPC value of 2.5. No BR symptoms were recorded on ‘Merlot’ bunches (**Figure 2**).

In 2021, the season was cool and dry, with an average temperature of 23.1°C (min = 14°C, max = 29.9°C), an average RH of 64%, a total of 167 mm of rain on 27 rainy days, and a total of 160 h of leaf wetness evenly distributed from May to August (**Figure 1D**). The phenological stages of the ‘5^th^ leaf unfolded’, full bloom, and veraison were recorded on ‘Merlot’ on May 12, June 10, and August 5, respectively; berry ripening occurred at the beginning of September. Although the DM symptoms were first observed on ‘Merlot’ in mid-June, the disease did not further increase until mid-July (**Supplementary Figure S4**), and reached a final AUDPC value of 14.89 on leaves and 1.84 on bunches (**Figure 2**). PM epidemics began in early June (**Supplementary Figure S4**) and increased over time, reaching a final AUDPC value of 21.55 on leaves and 20.44 and bunches (**Figure 2**). BR symptoms were mainly observed in July and August (**Supplementary Figure S4**), with higher values on bunches (4.91) than on leaves (0.26; **Figure 2**).

GM was found only on bunches and only at very low levels, irrespective of the year or variety, probably because weather conditions were mostly dry during berry ripening (**Figure 1**). This disease was therefore excluded from further analyses.

### Disease progress on leaves vs. bunches

**Figure 3:**
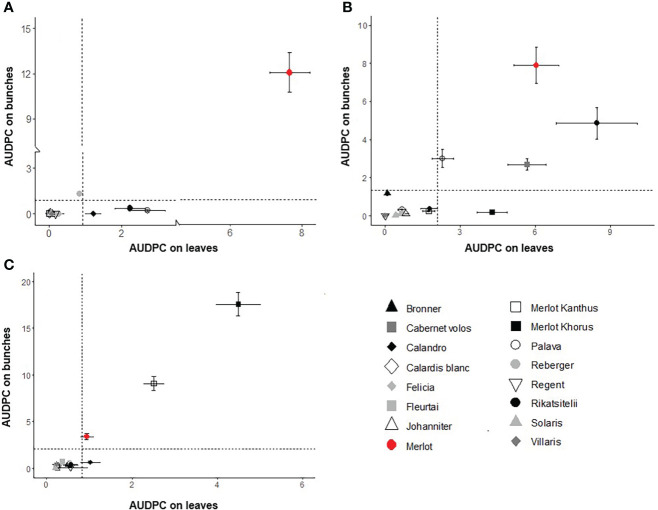
Scatter plots of AUDPC on leaves and bunches for downy mildew **(A)**, powdery mildew **(B)**, and black-rot **(C)** for the grape varieties listed in **Table 1**. Dots represent the average of a 4-year period; whiskers represent the standard error. Dotted lines dividing the graph into four quadrants indicate the 4-year average of the AUDPC values calculated for all varieties on leaves and bunches. The upper right quadrant includes varieties with high disease on both leaves and bunches; the lower right quadrant includes varieties with high disease on leaves and low disease on bunches; the upper left quadrant includes varieties with low disease on leaves and high disease on bunches; and the lower left quadrant includes varieties with low disease on both leaves and bunches.

**Figure 4:**
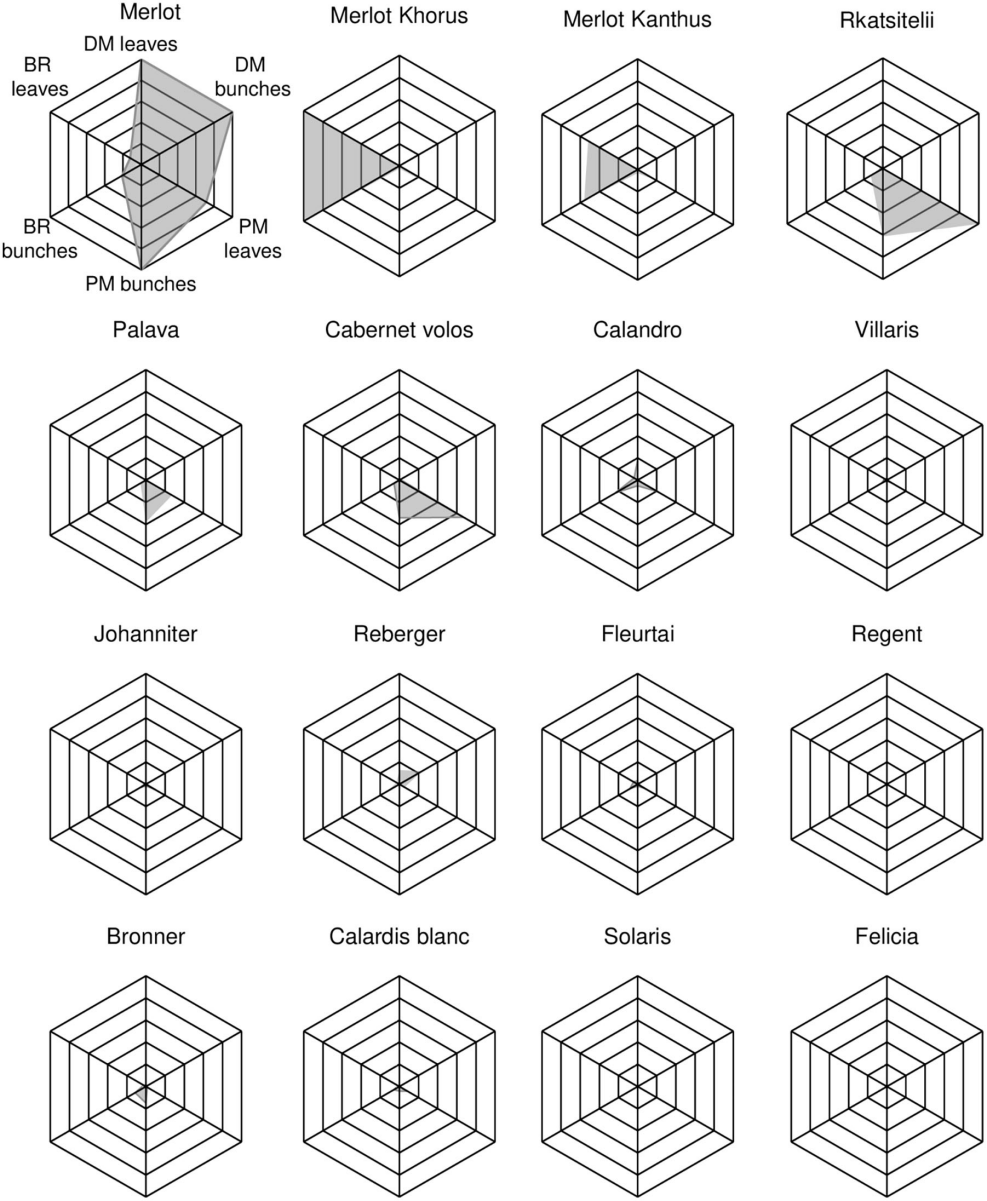
Spider graphs representing the resistance pattern of grapevine varieties with regard to downy mildew (DM), powdery mildew (PM), and black rot (BR) on leaves and bunches. In each spider graph, the black net ranges from 0 to 1 for each disease and organ, where 1 is the highest disease value found in the experiment, expressed as AUDPC (Area Under the Disese Progress Curve), which was 6.542 (‘Merlot’), 8.461 (‘Rkatsitelii’), 4.492 (‘Merlot Khorus’) on leaves for DM, PM, and BR, respectively, and 5.610 (‘Merlot’), 5.720 (‘Merlot’), and 17.583 (‘Merlot Khorus’) on bunches for DM, PM, and BR, respectively. The grey area is originated by the 4-year averages (also rescaled from 0 to 1) of AUDPC values. For each disease and organ, the rescaled 4-year average of AUDPC was obtained by dividing the value by the highest average AUDPC.

Scatter plots showing the relationships between AUDPC values on bunches vs. leaves for DM, PM, and BR are shown in **Figures 3A–C**, respectively. This information is summarized for each variety in the spider graphs shown in **Figure 4**. Disease progress curves on leaves and bunches for each variety are shown in details in the **Supplementary Material**.

For DM, the quadrants of the scatter plot were divided at AUDPC = 0.92 and 0.87, i.e., the 4-year average values on leaves and bunches, respectively (Fig 3A). The sensitive control ‘Merlot’ had high AUDPC values on both leaves and bunches. ‘Palava’ and ‘Rkatsitelii’, which do not have Rpvs, and ‘Calandro’, which has the locus Rpv3^1^, had high disease on leaves and low disease on bunches. ‘Reberger’, which does not have Rpvs, had low disease on leaves but high disease on bunches. All others varieties had low disease on both leaves and bunches.

For PM, the quadrants were divided at AUDPC = 2.09 (leaves) and 1.33 (bunches) (**Figure 3B**). ‘Merlot’, ‘Rkatsitelii’, ‘Palava’, and ‘Cabernet volos’, which do not have Ren loci, expressed higher sensitivity to PM on both leaves and bunches than the average the AUDPC values for all varieties. ‘Merlot Khorus’ had high levels of disease on leaves and low levels on bunches. All other varieties had low levels of PM and have Ren loci, with the exception of ‘Merlot Kanthus’ for which the presence of Ren loci remains to be determined.

For BR, the mean AUDPC values that divided the quadrants were = 0.82 and 2.14 on leaves and bunches, respectively (**Figure 3C**). The area corresponding to high sensitivity on both leaves and bunches included ‘Merlot’, ‘Merlot Khorus’, and ‘Merlot Kanthus’. For ‘Calandro’, sensitivity was higher on leaves and low on bunches than the average the AUDPC values for all varieties. All others varieties had low levels of disease on both leaves and bunches. No variety had high sensitivity on bunches and low sensitivity on leaves.

### Grouping of varieties based on the three diseases and definition of resistance patterns

**Figure 5:**
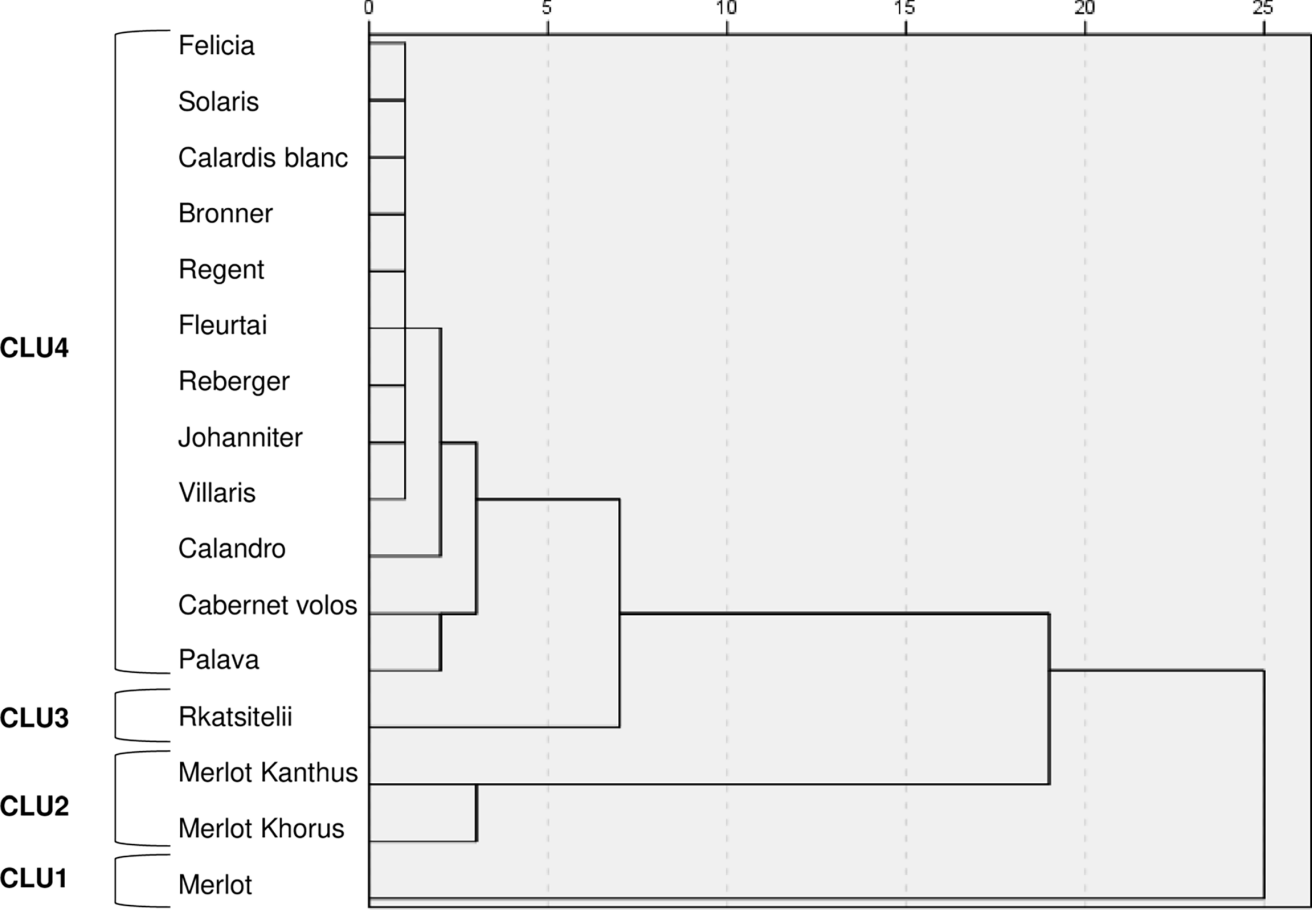
Dendrogram resulting from a hierarchical cluster analysis of the AUDPC values on leaves and bunches for downy mildew, powdery mildew, and black-rot in the 4-year period. CLU1 to CLU4 represent the four clusters at 5-unit dissimilarity cut-off point.

At a 5-unit dissimilarity cut-off point, the hierarchical cluster analysis grouped the varieties into four clusters (CLU1 to CLU4, **Figure 5**), which had significantly different AUDPC values (with P ranging from 0.04 to P< 0.001) (**Table 2**). Based on the LSD test, the varieties belonging to a cluster were considered to have high resistance, intermediate resistance, and low resistance to DM, PM, and BR (**Table 2**).

**Table 2:** Average values of AUDPC (area under the disease progress curve) for each disease on leaves and bunches for the four clusters of grapevine varieties shown in **Figure 5**.

Cluster	Downy mildew			Powdery mildew			Black-rot		
**CLU1**	9.355	a^2^	LR^3^	5.655	a	LR	1.548	b	IR
**CLU2**	0.020	c	HR	1.576	ab	IR	8.418	a	LR
**CLU3**	1.298	b	IR	5.849	a	LR	0.476	bc	IR
**CLU4**	0.303	c	HR	0.777	b	HR	0.335	c	HR
**P-value^1^**	< 0.001			0.04			< 0.001		

^1^ P-values indicate the significance level of the effect of clusters in an ANOVA carried out on ln-transformed AUDPC values.

^2^ Averages followed by the same letter in the same column are not significantly different according to Fisher-protected LSD test at P = 0.05.

^3^ Resistance level assigned to each cluster of grapevine varieties based on AUDPC values and the LSD test: HR, high resistance; IR, intermediate resistance; LR, low resistance. AUDPCs were calculated by using disease severity assessments carried out in the field in 2017, 2018, 2019, and 2021, on both leaves and bunches.

CLU1 included only the control ‘Merlot’; CLU1 was characterised by low resistance to DM and PM, and intermediate resistance to BR (**Table 2**). CLU2 included ‘Merlot Khorus’ and ‘Merlot Kanthus’ and was characterized by high resistance to DM, intermediate resistance to PM, and low resistance to BR; these varieties have one Rpv locus (**Table 1**), but the presence of Ren loci in their genome is uncertain. CLU3 included only ‘Rkatsitelii’, which lacks Rpv and Ren loci, and was characterized by intermediate resistance to DM and BR, and low resistance to PM. CLU4 included ‘Bronner’, ‘Felicia’, ‘Solaris’, ‘Calardis blanc’, ‘Regent’, ‘Reberger’, ‘Johanniter’, ‘Villaris’, ‘Calandro’, ‘Cabernet volos’, ‘Palava’, and ‘Fleurtai’; CLU4 was characterized by high resistance to DM, PM, and BR. All CLU4 varieties have 1 or 2 Rpv loci with the exception of ‘Palava’ and ‘Reberger’, and 1 to 3 Ren loci with exception of ‘Cabernet volos’ and ‘Palava’ (**Table 1**).

Considering all of the varieties together, the correlation coefficients between AUDPCs on leaves and bunches were significant (P<0.01), with r = 0.890 for DM, r = 0.786 for PM, and r = 0.986 for BR. The correlation coefficients calculated within each cluster, however, indicated that the leaf-to-bunch relationship was not consistent over the three resistance levels (**Table 3**). For DM, there was a significant (P = 0.006) relationship between the AUDPC on leaves and bunches only for CLU 1 (‘Merlot’), indicating that low resistance is expressed by both leaves and bunches with r = 0.763. For the varieties in CLU 2 to CLU 4 (which have an intermediate to a high level of DM resistance), in contrast, the disease progress of DM on leaves and bunches was not significantly correlated (P > 0.01). A similar relationship was observed for BR, i.e., the resistance expressed by leaves and bunches was significantly correlated (r = 0.621; P = 0.001) only for CLU 2 (low resistance). For PM, the relationship was significant for all of the clusters with low and intermediate resistance (CLU 1 to CLU 3) but not for the cluster (CLU4) with high resistance.

**Table 3:** Correlation coefficients between the AUDPC (area under the disease progress curve) values assessed on leaves and bunches for the grapevine varieties belonging to each of the four clusters shown in **Figure 5** and **Table 2**.

Cluster	Downy mildew		Powdery mildew		Black-rot	
**CLU1**	LR^1^	0.763^2^	LR	0.958	IR	0.166
		*0.006* (12)^3^		*<0.001* (12)		*0.607* (12)
**CLU2**	HR	0.018	IR	0.601	LR	0.621
		*0.933* (24)		*0.002* (24)		*0.001* (24)
**CLU3**	IR	0.225	LR	0.992	IR	0.515
		*0.592* (8)		*<0.001* (8)		*0.296* (8)
**CLU4**	HR	0.215	HR	0.138	HR	0.226
		*0.017* (122)		*0.139* (122)		*0.012* (122)

^1^ Resistance level assigned to each cluster of grapevine varieties based on AUDPC values and the LSD test: HR = high resistance, IR = intermediate resistance, LR = low resistance (see **Table 2**).

^2^ Pearson’s coefficients of correlation between AUDPC values assessed on leaves and bunches.

^3^ P-value (in italics) and number of cases (between brackets) on which the correlation coefficients are based. AUDPCs were calculated by using disease severity assessments of both leaves and bunches in the field in 2017, 2018, 2019, and 2021. The P-value is shown for each correlation coefficient.

## Discussion

The present research characterised the resistance pattern of 16 grape varieties to four main grape pathogens, i.e., *P. viticola, E. necator, B. cinerea*, and *P. ampelicida*. Most of 16 varieties carry one or more Rpv loci conferring partial resistance to DM and/or Ren loci conferring partial resistance to PM. Resistance was assessed in the field and relative to a susceptible control, the variety ‘Merlot’, over four grape-growing seasons (2017, 2018, 2019, and 2021). The conduciveness of environmental conditions for the diseases differed among the four growing seasons such that epidemics of DM, PM, and BR developed on both leaves and bunches with varying severities, while GM affected only bunches and only at very low levels. Therefore, incomplete information was collected with respect to GM. Low levels of GM detected in this study are likely due to unfavourable weather conditions. Further studies are needed to investigate the resistance to GM of the varieties showing resistance to DM and/or PM.

In previous research, resistance to mildews was largely evaluated by performing monocycle, *in vitro* bioassays involving the artificial inoculation of leaf discs (Stein et al., 1985; Eibach et al., 1989; Staudt and Kassemeyer, 1995; Brown et al., 1999; Boso et al., 2006; Boso and Kassemeyer, 2008; Bellin et al., 2009); leaf disc inoculation has also been used to study the components of partial resistance to *P. viticola* in some resistant varieties (Bove and Rossi, 2020). Before the current study, little information was available about the behaviour of these resistant varieties under field conditions (Fischer et al., 2004; Wingerter et al., 2021), in which infection cycles follow each other during the season so that the resistance components related to sporulation (i.e., length of latency and infectious periods, sporulation rate, and infectiousness of zoosporangia; Bove and Rossi, 2020) may have effects that are undetected in monocycle leaf disc bioassays. For this reason, the whole epidemic course of each disease was considered and summarized by the AUDPC values in the current study. To our knowledge, this is the first research report of the field evaluation of resistance to DM, PM, and BR in resistant grapevine varieties expressed both on leaves and bunches under natural conditions of inoculum and without application of fungicides.

In this research, we introduce the term “resistance pattern” to describe the resistance level of a variety to all of the diseases occurring in a field experiment. This concept is similar to the term “resistance pattern” that is commonly used in bacteriology with respect to antibiotic resistance; based on the CDCs (Centers for Disease Control and Prevention) glossary of terms related to antibiotic resistance, the term describes the antibiotic resistance of a single isolate to multiple antibiotics.

Overall, our research divided the 16 grape varieties into four main groups (CLU1 to CLU4) based on the resistance pattern to the three diseases. The first group (CLU1) included only the control, ‘Merlot’, which had low resistance to mildews and intermediate resistance to BR. The second group (CLU2), made up of ‘Merlot Khorus’ and ‘Merlot Kanthus’, had high resistance to DM conferred by Rpv12 and Rpv3, respectively. ‘Merlot Khorus’ and ‘Merlot Kanthus’ share the same pedigree (Merlot x 20/3) and have been genetically studied only for Rpv loci, while the presence of Ren and Rgb loci is still uncertain. Given the results of the current study, it is unlikely that these varieties carry Rgbs; both varieties were severely affected by BR over the 4 years of the study, with AUDPC values on both leaves and bunches greater than those of the sensitive control, ‘Merlot’. CLU2 had intermediate resistance against PM, with *E. necator* colonies observed mainly on leaves rather than on bunches. The third cluster (CLU3) included only ‘Rkatsitelii’, a native Georgian variety that showed intermediate resistance to DM despite the absence of Rpv loci in its germplasm. The intermediate resistance of ‘Rkatsitelii’ to DM reflected the low level of resistance of its leaves and the high level of resistance of its bunches during the four growing seasons (**Figure 3A**). ‘Rkatsitelii’ also had intermediate resistance to BR, with its leaves less resistant to the disease than its bunches (**Figure 3C**). ‘Rkatsitelii’ had high levels of PM on both its leaves and bunches, with AUDPC values on its leaves much greater than those on the leaves of the control, ‘Merlot’. The fourth cluster (CLU4), which included the majority of the investigated varieties, showed high resistance to the three diseases. On these varieties, DM, PM, and BR symptoms were rarely observed on leaves, and bunches remained generally healthy over the four growing seasons.

Among the four cluster, only CLU1 had low resistance to DM, meaning that all of the other varieties were resistant to DM to some extent under field conditions; these other DM-resistant varieties included ‘Rkatsitelii’ (CLU3), ‘Reberger (CLU4), and ‘Palava’ (CLU4), which lack Rpv loci in their germplasm. The expression of resistance to DM was generally highest in the varieties carrying Rpv3, Rpv10, or Rpv12; these observations were consistent with previous studies (Basler and Pfenninger, 2003; Pezet et al., 2004; Vezzulli et al., 2018; Bove and Rossi, 2020). In a recent study, the 16 varieties considered in the current research were characterized for six components of resistance (infection frequency, latent period, lesion size, production of sporangia, infectious period, and infectivity of sporangia) to DM by leaf disc inoculation under laboratory conditions (Bove and Rossi, 2020). The findings of the latter study were confirmed by our observations. For example, ‘Merlot Kanthus’, ‘Johanniter’, ‘Bronner’, ‘Solaris’, ‘Calardis blanc’, ‘Merlot Khorus’, ‘Cabernet volos’, ‘Villaris’, and ‘Fleurtai’ showed high resistance to DM in both the laboratory assays (Bove and Rossi, 2020) and in the field experiment of the current study. In contrast, ‘Felicia’ and ‘Regent’ had low levels of resistance to DM in the laboratory (Bove and Rossi, 2020) but high levels of resistance in the field (current study). Although their AUDPC values were much lower, especially on bunches, than those of ‘Merlot’, the varieties ‘Calandro’, ‘Palava’, ‘Rkatsitelii’, and ‘Reberger’ were less resistant to DM in the current study than the previously mentioned resistant varieties, which was consistent with other studies (Boso and Kassemeyer, 2008; Bitsadze et al., 2015; Bove and Rossi, 2020).

Discrepancies between laboratory studies (conducted with high disease pressure and optimal environmental conditions for *P. viticola*) and field observations have been already reported (Calonnec et al., 2013; Vezzulli et al., 2018), and may be caused by differences in inoculum dose and environmental conditions, as well as by the co-occurrence of multiple diseases and the consequent competition among pathogens for the infection sites.

In studies that evaluate the resistance against a specific disease, all other diseases are usually controlled by the application of fungicides that do not affect the disease being studied. In the current research, however, no fungicides were applied during the growing season, so that the different pathogens were competing for the same infection sites. If we consider the crop as a finite collection of infection sites with two mutually exclusive states, healthy or infected (Savary et al., 2018), and if we assume that a site can be occupied by only one pathogen, then the occurrence of one disease limits the occurrence of the others. This could explain the intermediate level of resistance to DM of ‘Rkatsitelii’, which differs from the previous findings of Bove and Rossi (2020) and Bitsadze et al. (2015), who rated this variety as having low resistance to DM. In the current study, PM severely affected ‘Rkatsitelii’ and may have reduced the available sites for infection by *P. viticola*, resulting in an overestimation of the resistance to DM for this variety. This “competition-for-infection sites” explanation may also be applied to ‘Merlot Khorus’ and ‘Merlot Kanthus’, which were severely affected by BR over the four growing seasons. This may have reduced infections by PM and DM, leading to an overestimation of the effective level of resistance against these two diseases. Nevertheless, these two varieties showed high resistance to DM in leaf disc inoculation tests (Bove and Rossi, 2020). Pathogen-specific *in vitro* studies with ‘Merlot Khorus’ and ‘Merlot Kanthus’ are needed to better characterize their level of resistance to PM; because the presence of Ren loci in these two varieties is still uncertain, genetic profiling is also needed.

The results of the current study increase our understanding on the resistance of grape bunches to diseases. When all the varieties were considered, the positive correlation between the resistance of leaves vs. bunches for all three diseases seems to support the results of Calonnec et al. (2013) on the reliability of bioassays on leaves for predicting disease severity on bunches. However, when the correlations were assessed for groups of varieties showing a similar degree of resistance, the correlation between the AUDPC on leaves and bunches was significant only when the resistance level was low, with the exception of PM. We therefore conclude that the bioassays on leaves can indicate the level of resistance of bunches in the field for susceptible varieties but not for resistant ones.

More than 70% of the fungicide used in Europe is applied to vineyards (Muthmann and Nadin, 2007). According to the Directive 2009/128/EC, the use of plant genotypes carrying resistance genes to major grape diseases may help to reduce the application of plant protection products and thus the negative effects of those products on the environment and human health and may also reduce vineyard production costs. Although some studies have shown that resistant grapevine varieties can reduce the application of fungicides by 100% (Wingerter et al., 2021), other studies indicate that resistant varieties still require the application of biocides to protect against certain oomycete and fungal pathogens (Töpfer et al., 2011; Delmas et al., 2016). By defining the resistance patterns of 15 resistant varieties to three grapevine diseases (DM, PM, and BR), our study provides a more nuanced and more useful perspective than that provided by single-pathogen laboratory studies on the resistance of these varieties under field conditions. Information on the resistance patterns could support both the strategic and tactical decisions of grape disease management (Rossi et al., 2012; Pertot et al., 2017; Rossi et al., 2019).

At the strategic level, an understanding of resistance patterns may help growers select the variety to be planted in a specific environment. For example, in a location with frequent spring rains and high moisture, i.e., a location where PM rather than DM and BR prevails, a variety showing high or intermediate resistance to *E. necator* would be preferred such as varieties grouped in CLU2 and CLU4 in the current research. On the other hand, in locations where DM and BR are favoured, varieties of CLU4 should be preferred. The planting of varieties grouped in CLU1 and CLU3 should be carefully considered in locations with a history of severe grapevine diseases, especially in organic farms in which the use of synthetic fungicides is banned.

At the tactical level, if a variety expresses high resistance to DM and PM but high susceptibility to BR, intensive control of *P. ampelicida* but light control of *P. viticola* and *E. necator* would be required. This could be the case of ‘Merlot Khorus’ and ‘Merlot Kanthus’ in CLU2. For the varieties grouped in CLU4, tactical disease management can require few fungicide applications, which can be scheduled using an infection risk-based approach. For this purpose, information on resistance patterns could be used to modify mathematical models so that they provide variety-specific predictions of disease risk. These models usually predict the risk of infection based on the weather conditions and, in some cases, the growth stage of the host (Rossi et al., 2008; Caffi et al., 2011; González-Domínguez et al., 2015; Rossi et al., 2015; Brischetto et al., 2021), but they do not presently use information concerning the resistance level of the variety. The usefulness of such models to growers would be increased if they include resistance patterns and thereby provide more precise information on whether and when a fungicide treatment would be needed for any grape variety.

## Data availability statement

The raw data supporting the conclusions of this article will be made available by the authors upon request, without undue reservation.

## Author contributions

VR mainly contributed to the conception and the design of the study. IS and FB carried out the data collection in field. All authors contributed to the analysis of results and collaborated in writing the manuscript. All authors contributed to the article and approved the submitted version.

## Funding

This research was funded by research fundings of Università Cattolica del Sacro Cuore.

## Acknowledgments

The authors thank Gultekin Hasanaliyeva and Othmane Taibi (Università Cattolica del Sacro Cuore) for their valuable assistance in data collection in field.

## Conflict of interest

Author FB was employed by the company Horta srl.

The remaining authors declare that the research was conducted in the absence of any commercial or financial relationships that could be construed as a potential conflict of interest.

## Publisher’s note

All claims expressed in this article are solely those of the authors and do not necessarily represent those of their affiliated organizations, or those of the publisher, the editors and the reviewers. Any product that may be evaluated in this article, or claim that may be made by its manufacturer, is not guaranteed or endorsed by the publisher.
