# Supplementary material for: Information on disease resistance patterns of grape varieties may improve disease management
Source: Front Plant Sci. 2022 Nov 14;13:1017658. doi: 10.3389/fpls.2022.1017658 (PMC9704053; doi:10.3389/fpls.2022.1017658)
Supplement: Supplementary file 1 [file DataSheet_1.pdf]

Supplementary materials

The supplementary material contains details on disease progression on 15 partially resistant grapevine varieties and the positive control ‘Merlot’.

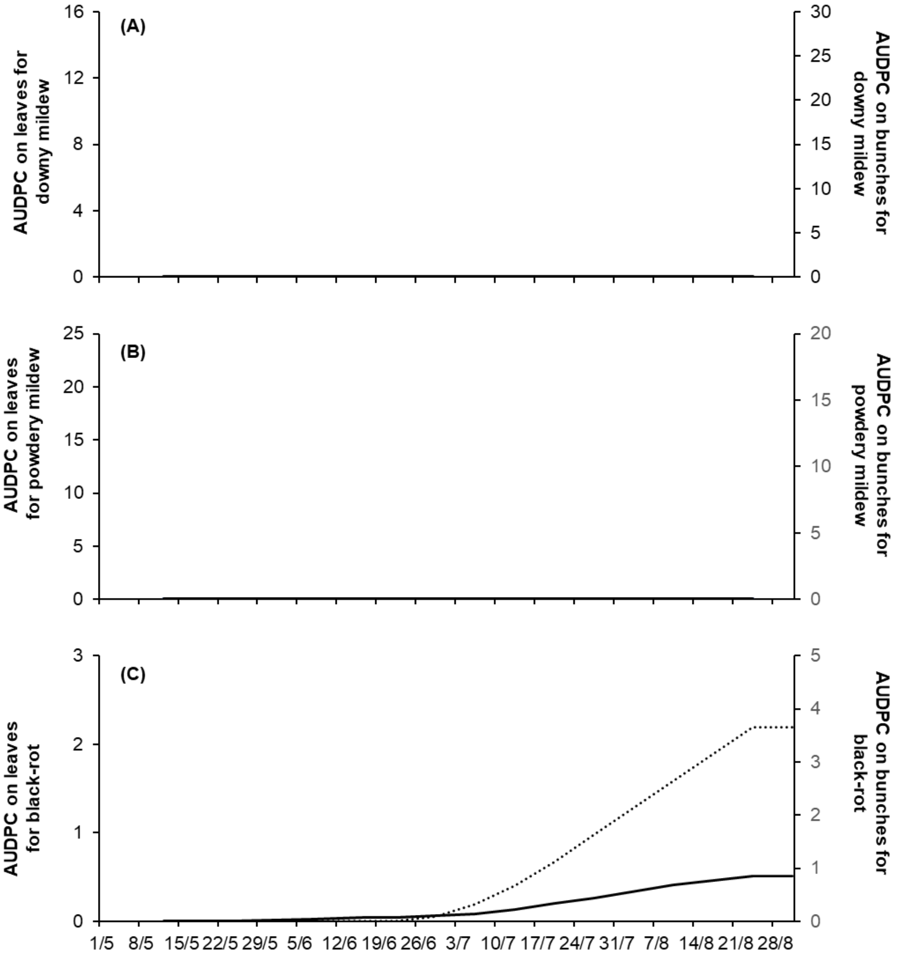

Figure S1. Data for AUDPCs recorded on the positive control ‘Merlot’ during 2017. (A) AUDPC values for downy mildew on leaves (solid line) and bunches (dotted line). (B) AUDPC values for powdery mildew on leaves (solid line) and bunches (dotted line). (C) AUDPC values for black-rot on leaves (solid line) and bunches (dotted line).

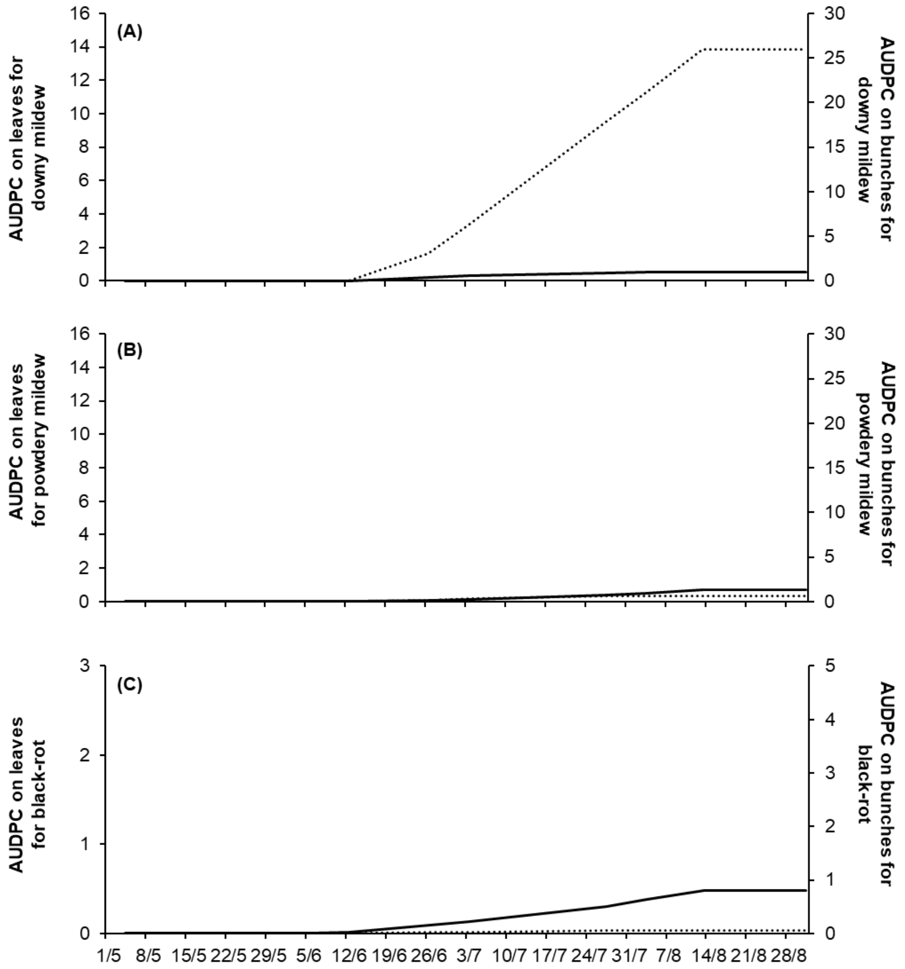

Figure S2. Data for AUDPCs recorded on the positive control 'Merlot' during 2018. (A) AUDPC values for downy mildew on leaves (solid line) and bunches (dotted line). (B) AUDPC values for powdery mildew on leaves (solid line) and bunches (dotted line). (C) AUDPC values for black-rot on leaves (solid line) and bunches (dotted line).

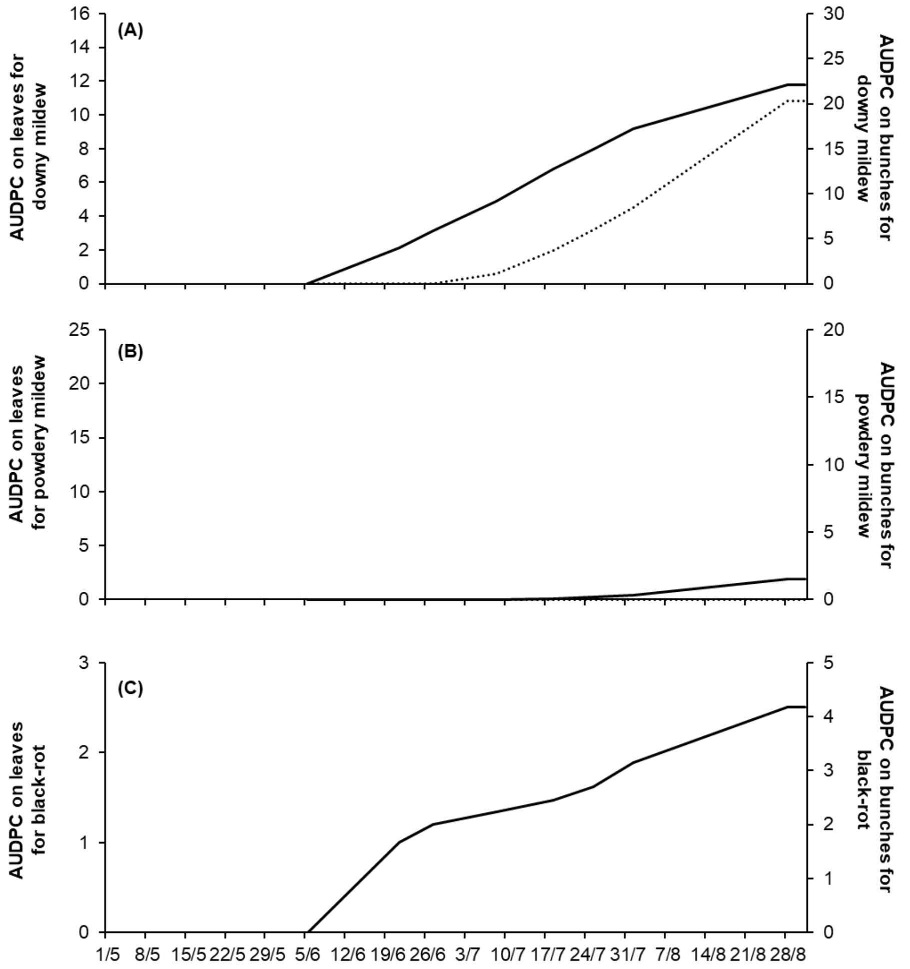

Figure S3. Data for AUDPCs recorded on the positive control 'Merlot' during 2019. (A) AUDPC values for downy mildew on leaves (solid line) and bunches (dotted line). (B) AUDPC values for powdery mildew on leaves (solid line) and bunches (dotted line). (C) AUDPC values for black-rot on leaves (solid line) and bunches (dotted line).

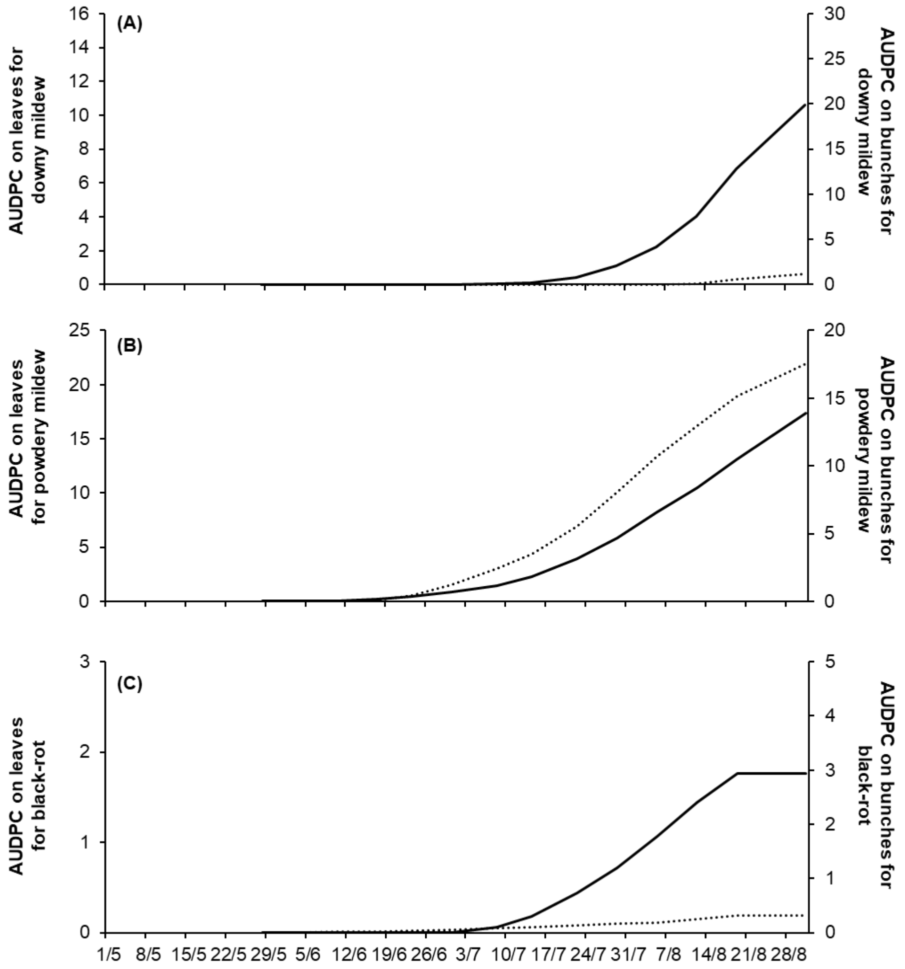

Figure S4. Data for AUDPCs recorded on the positive control 'Merlot' during 2021. (A) AUDPC values for downy mildew on leaves (solid line) and bunches (dotted line). (B) AUDPC values for powdery mildew on leaves (solid line) and bunches (dotted line). (C) AUDPC values for black-rot on leaves (solid line) and bunches (dotted line).

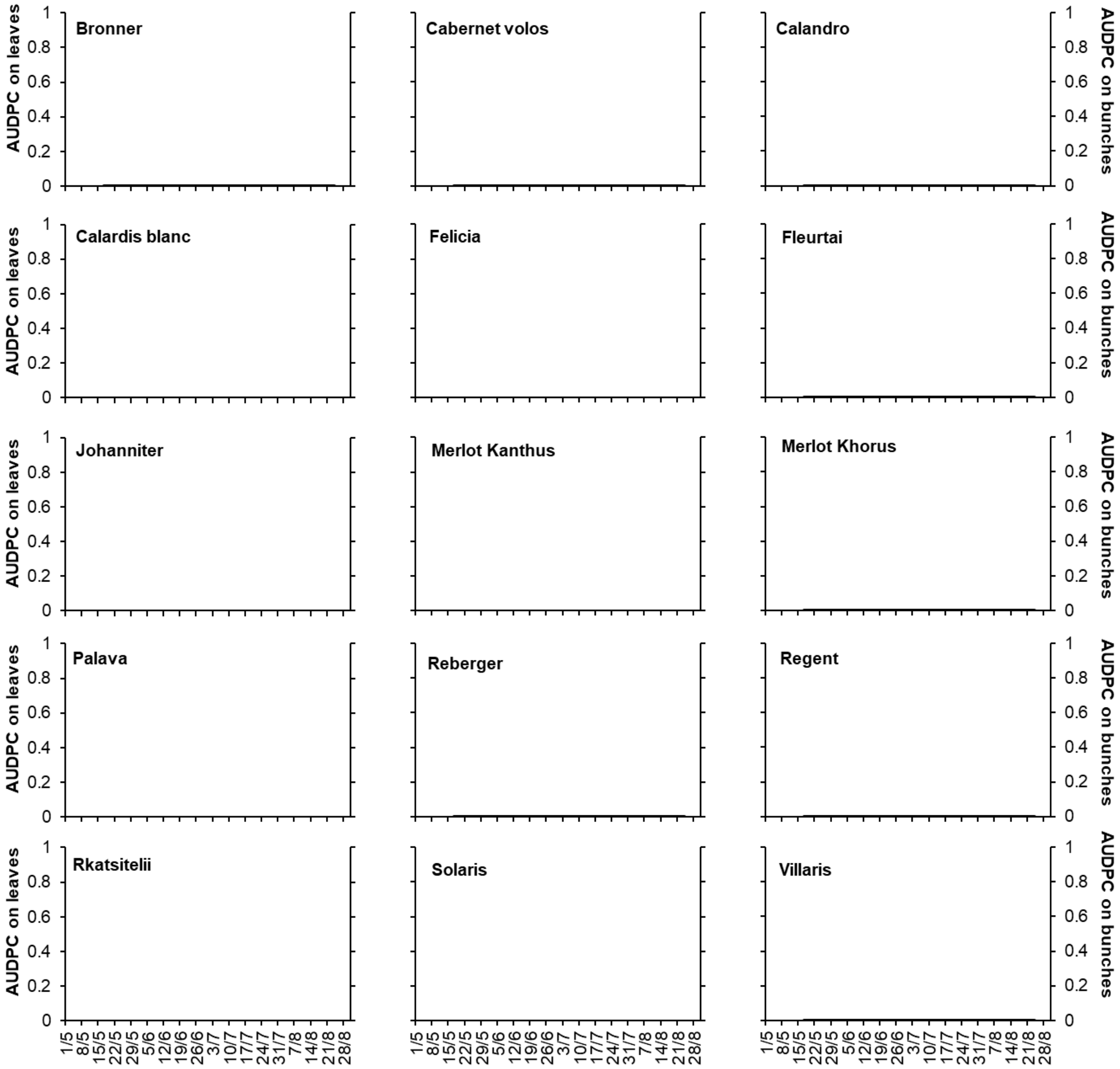

Figure S5. Data for AUDPCs for downy mildew on leaves (solid line) and bunches (dotted line) recorded on the partially resistant varieties during 2017.

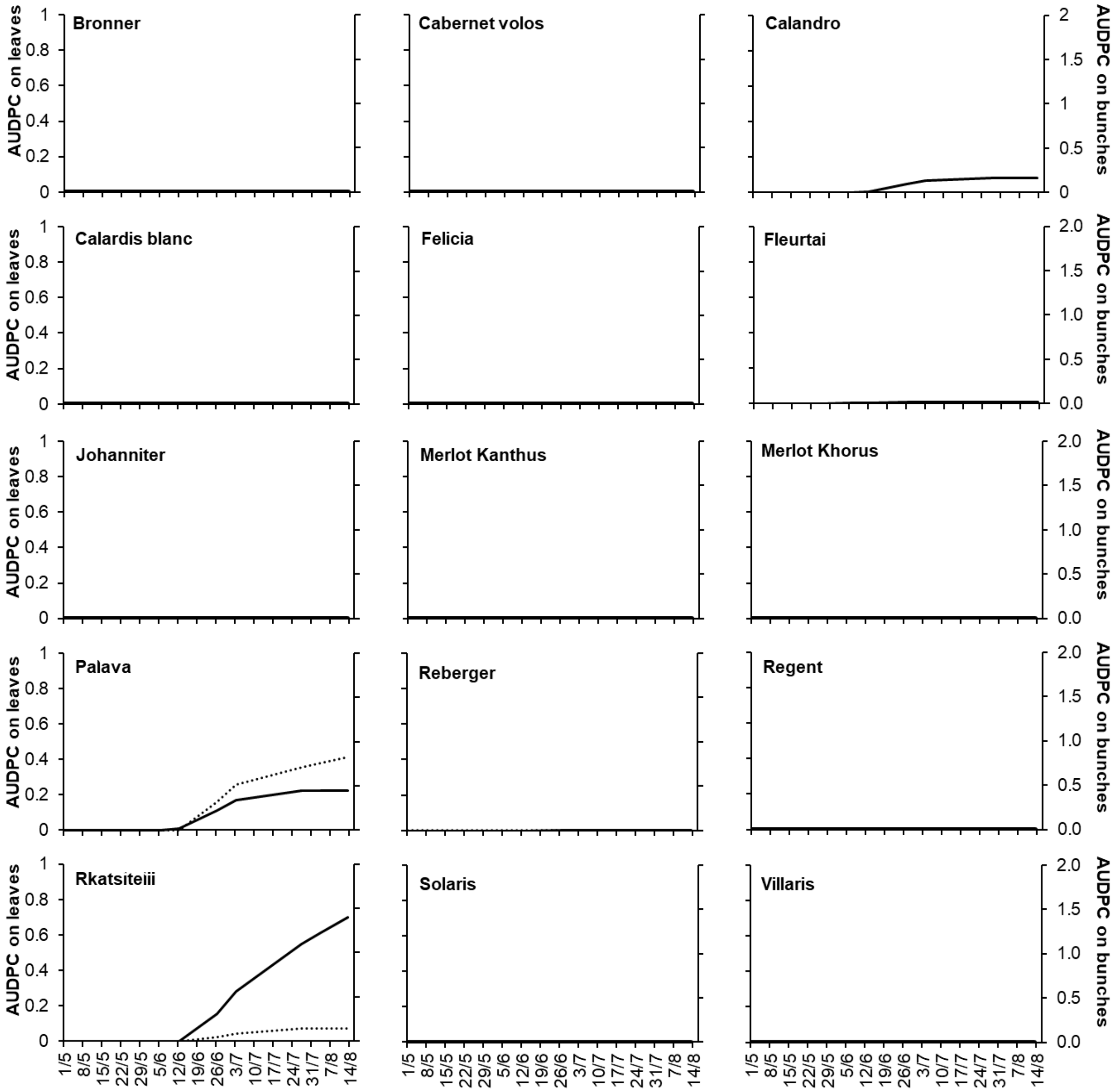

Figure S6. Data for AUDPCs for downy mildew on leaves (solid line) and bunches (dotted line) recorded on the partially resistant varieties during 2018.

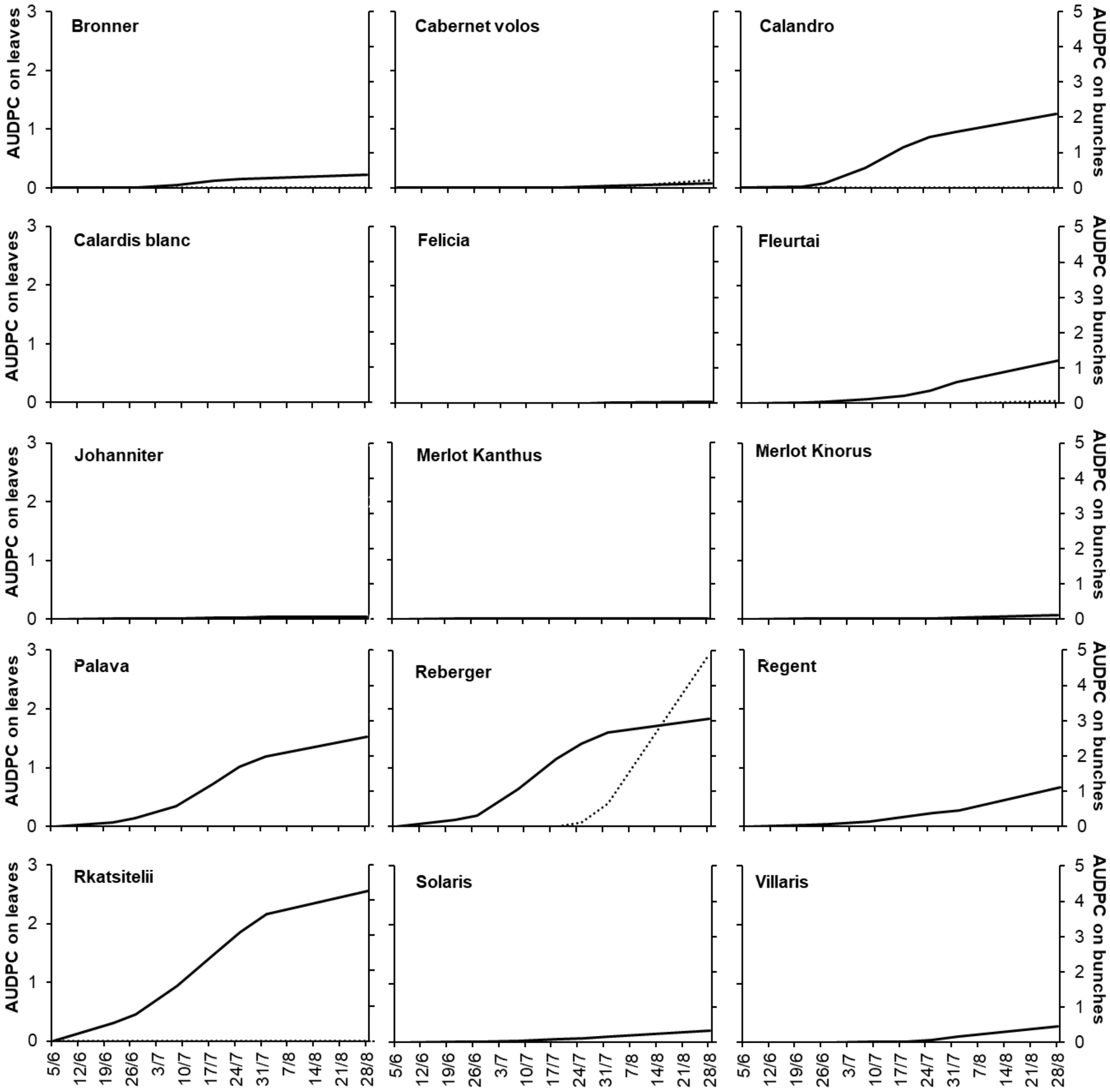

Figure S7. Data for AUDPCs for downy mildew on leaves (solid line) and bunches (dotted line) recorded on the partially resistant varieties during 2019.

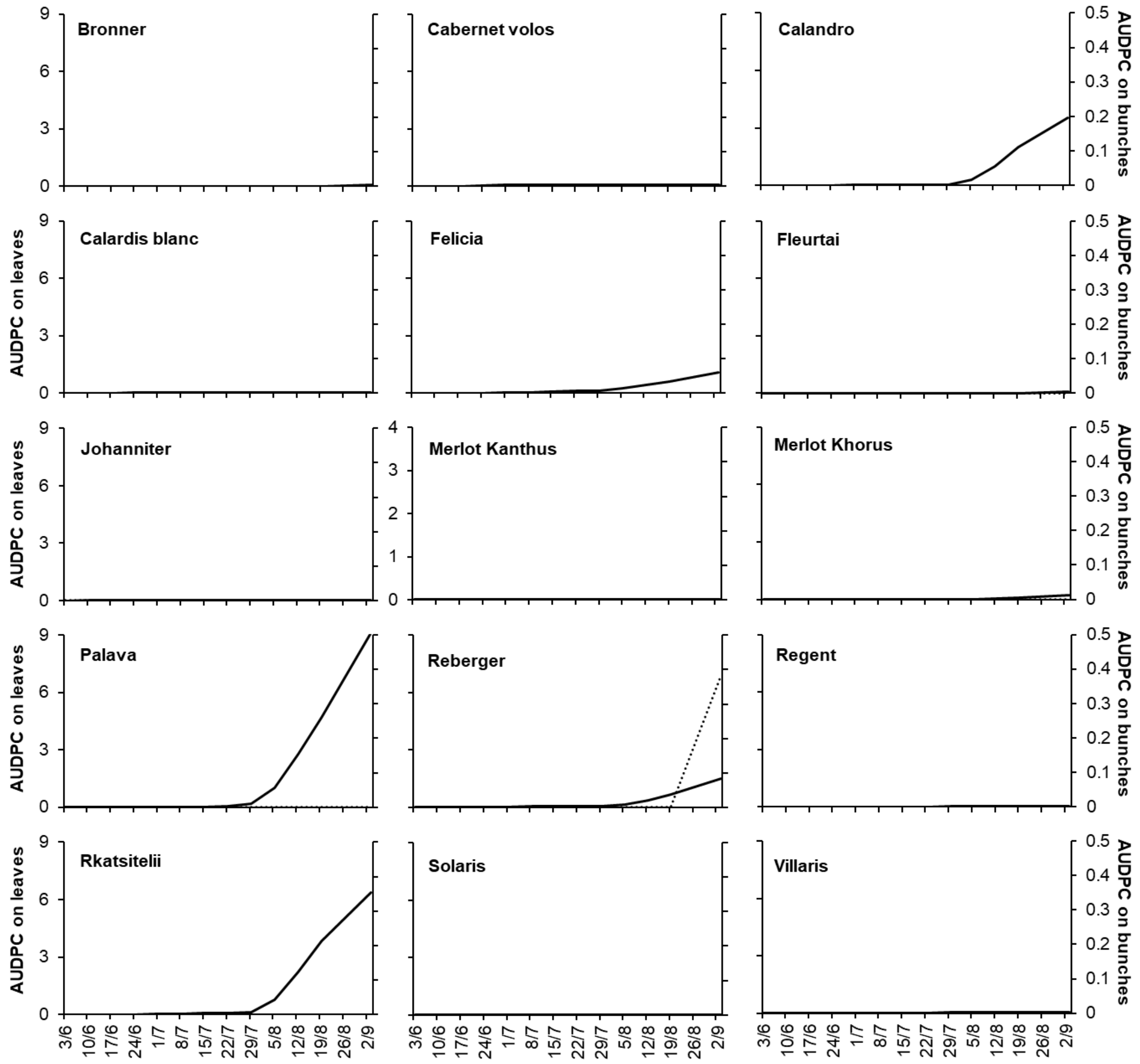

Figure S8. Data for AUDPCs for downy mildew on leaves (solid line) and bunches (dotted line) recorded on the partially resistant varieties during 2021.

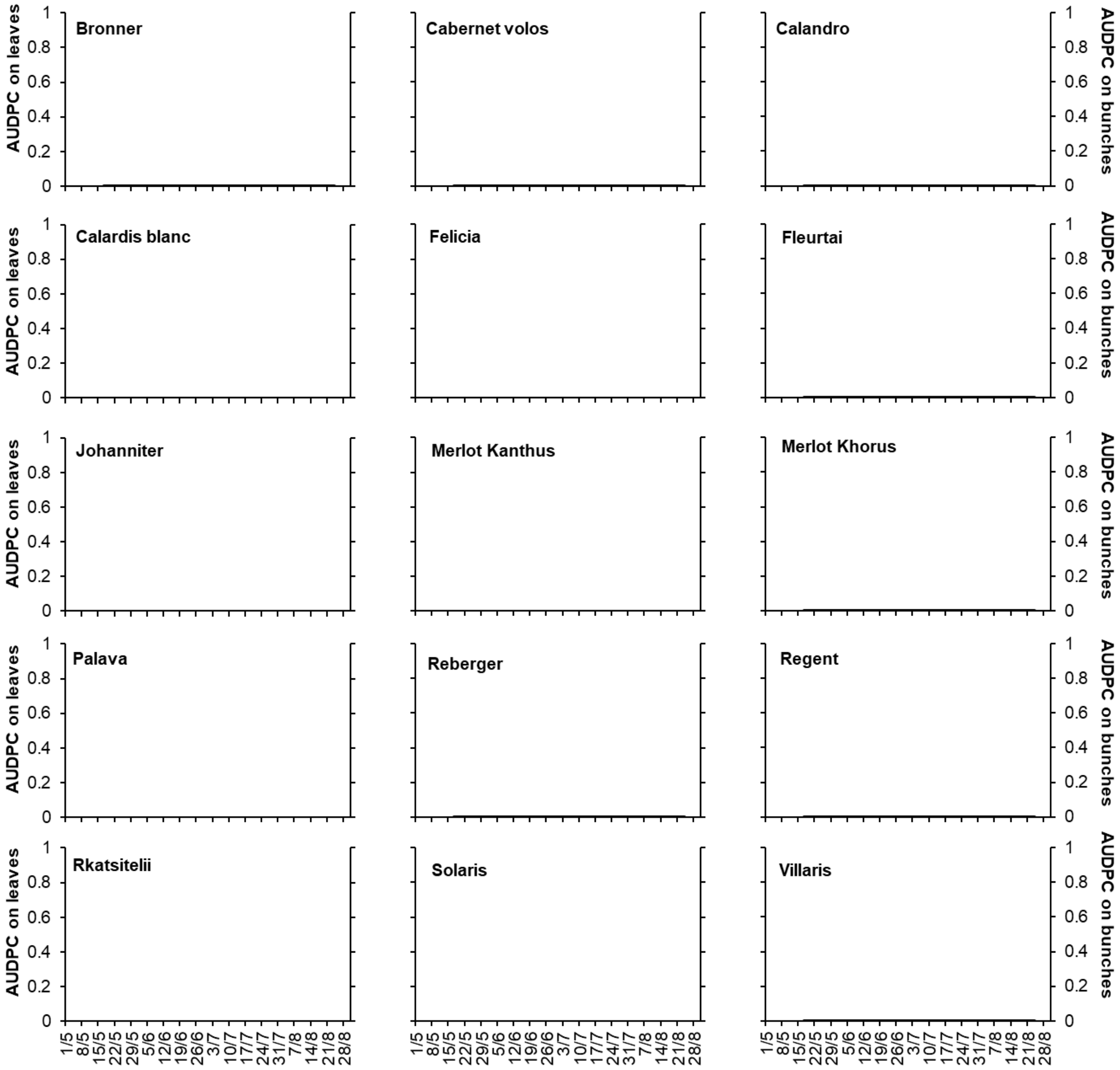

Figure S9. Data for AUDPCs for powdery mildew on leaves (solid line) and bunches (dotted line) recorded on the partially resistant varieties during 2017.

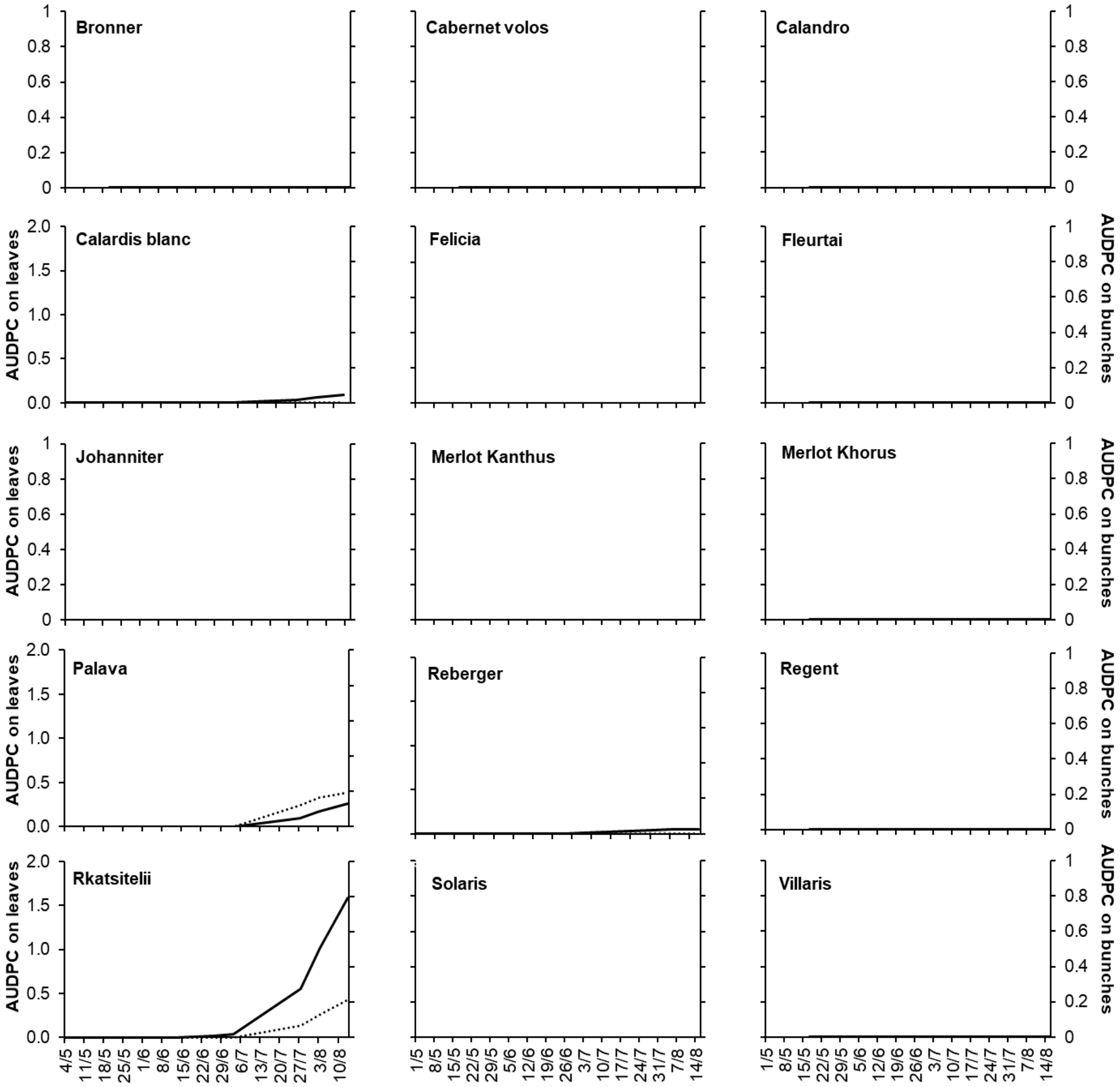

Figure S10. Data for AUDPCs for powdery mildew on leaves (solid line) and bunches (dotted line) recorded on the partially resistant varieties during 2018.

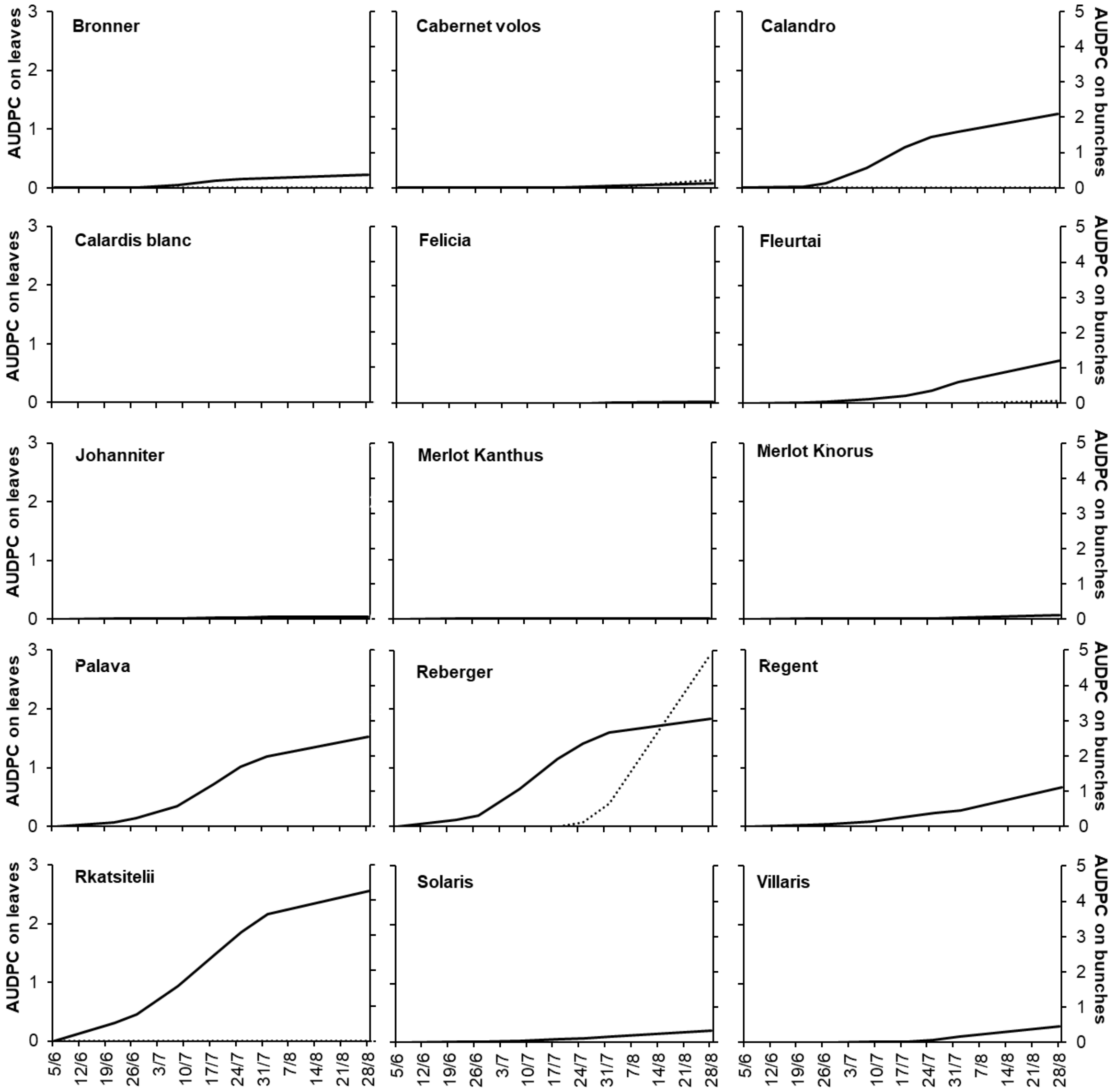

Figure S11. Data for AUDPCs for powdery mildew on leaves (solid line) and bunches (dotted line) recorded on the partially resistant varieties during 2019.

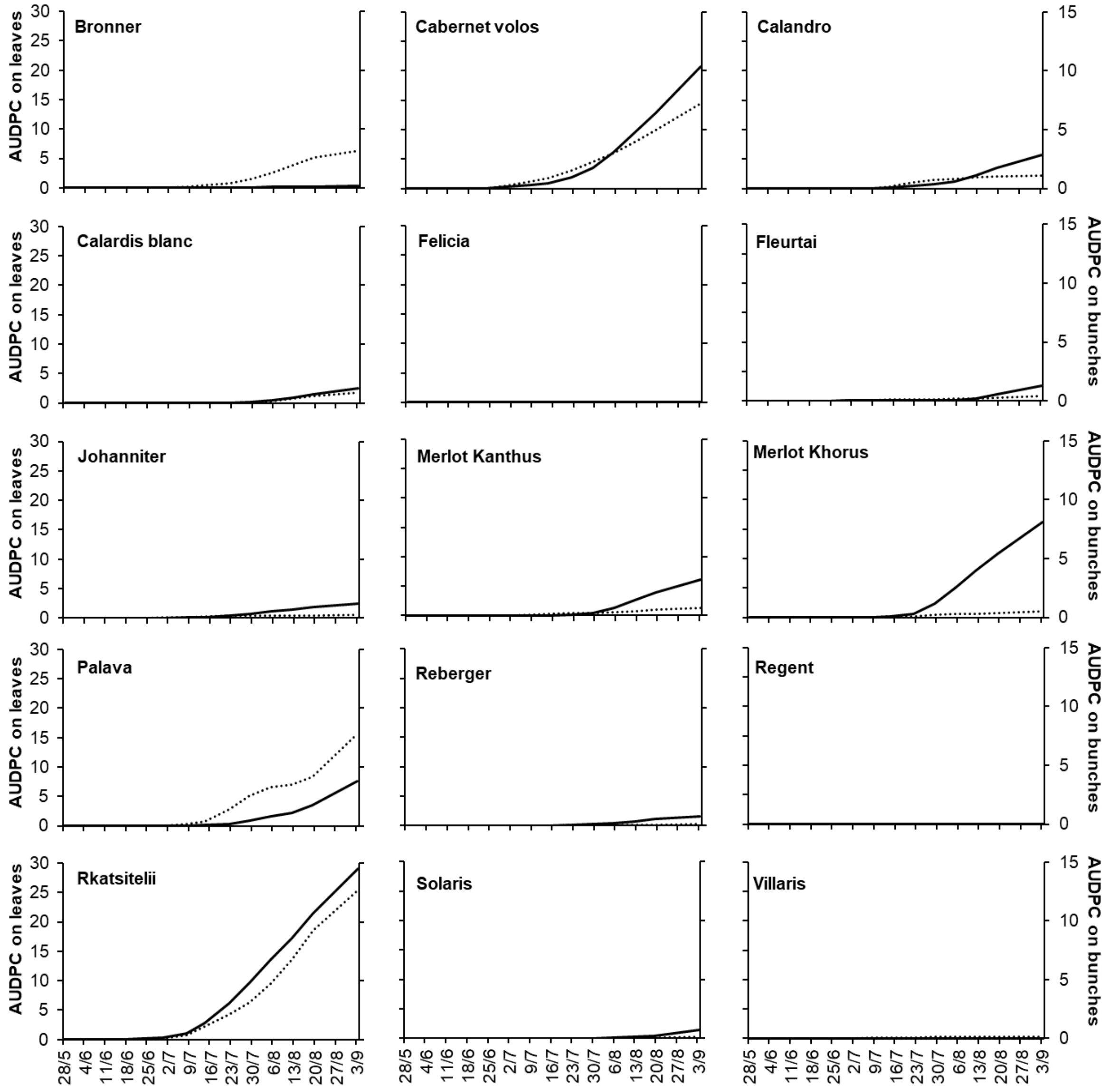

Figure S12. Data for AUDPCs for powdery mildew on leaves (solid line) and bunches (dotted line) recorded on the partially resistant varieties during 2021.

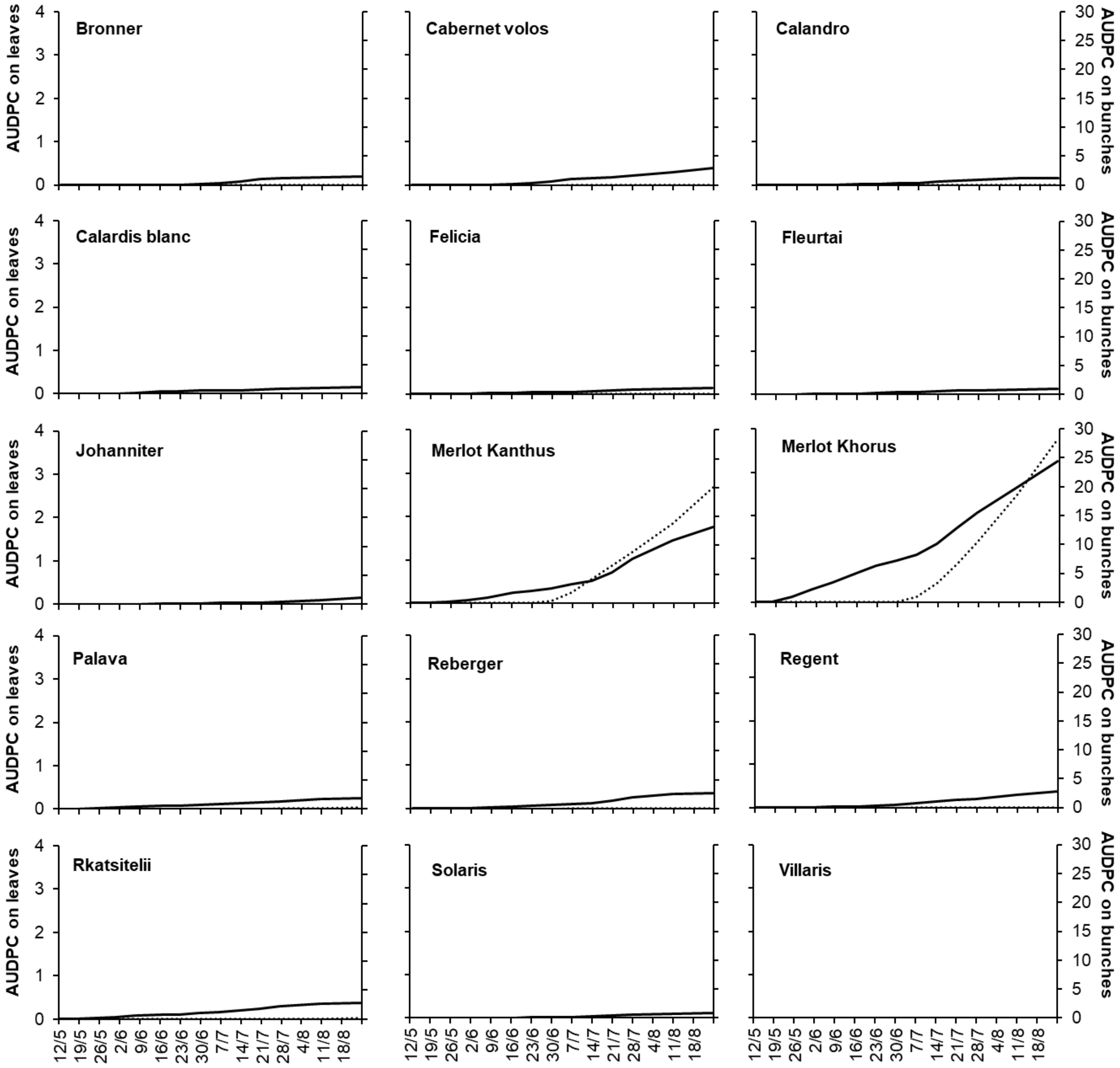

Figure S13. Data for AUDPCs for black rot on leaves (solid line) and bunches (dotted line) recorded on the partially resistant varieties during 2017.

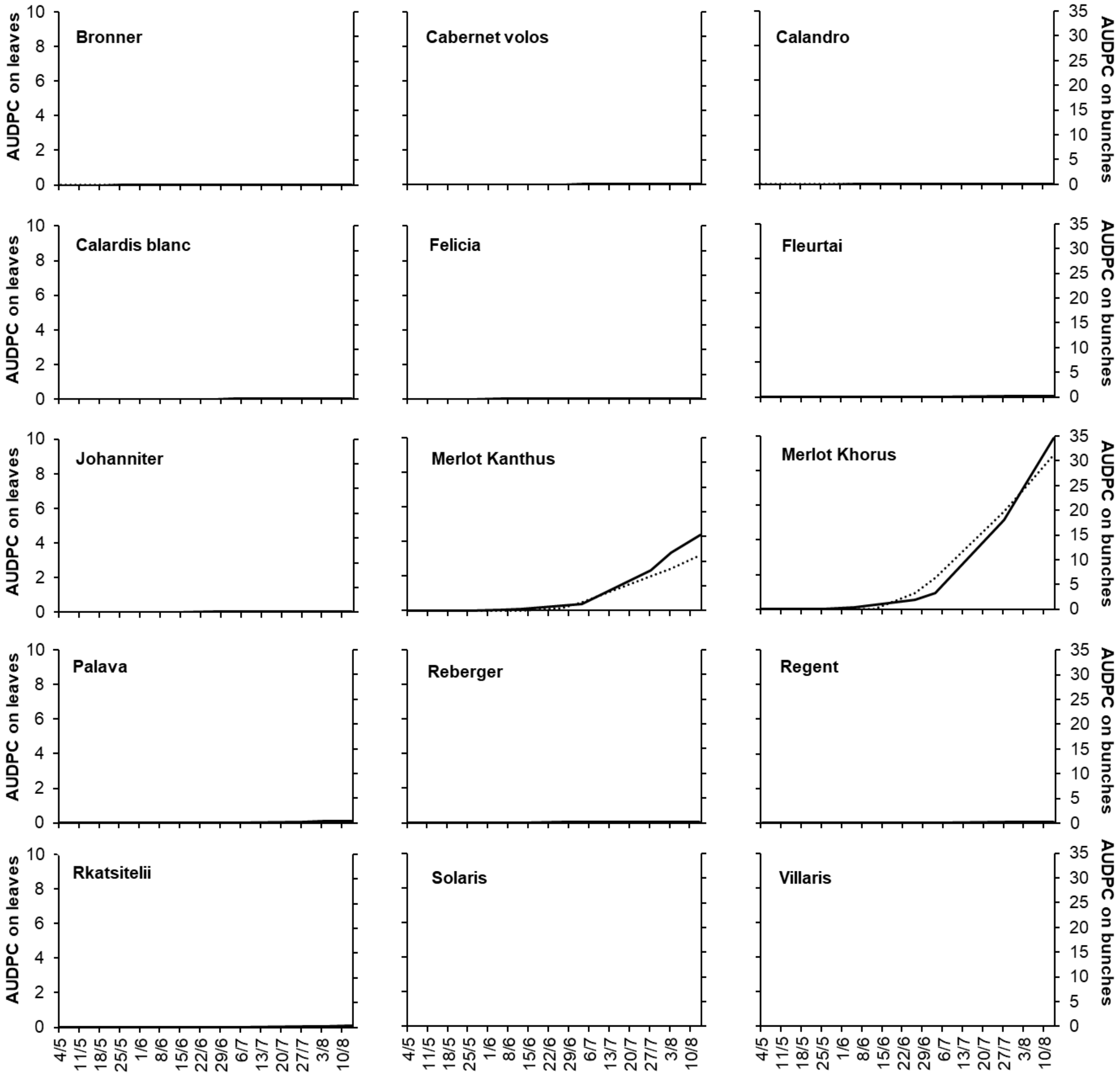

Figure S14. Data for AUDPCs for black rot on leaves (solid line) and bunches (dotted line) recorded on the partially resistant varieties during 2018.

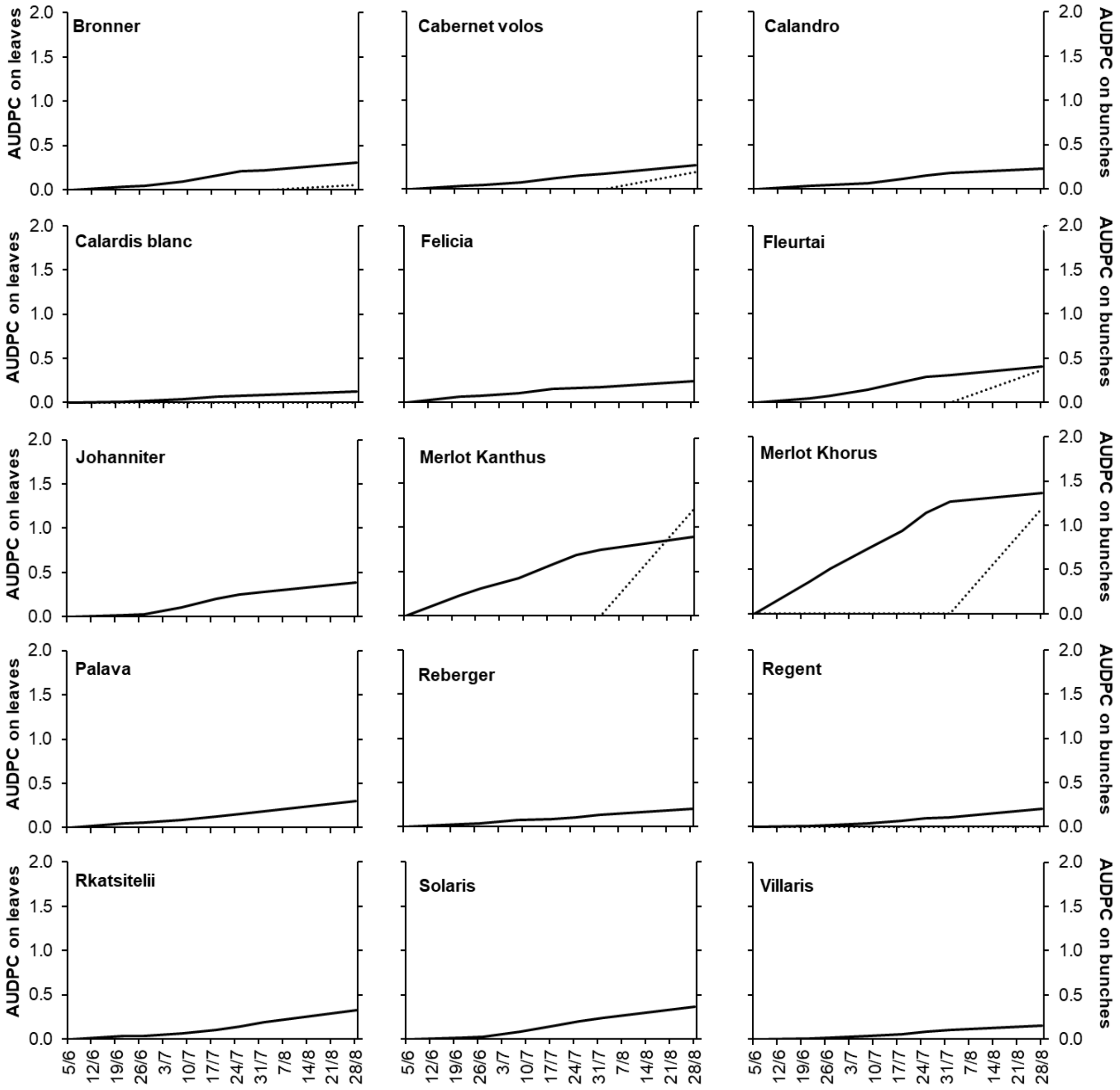

Figure S15. Data for AUDPCs for black rot on leaves (solid line) and bunches (dotted line) recorded on the partially resistant varieties during 2019.

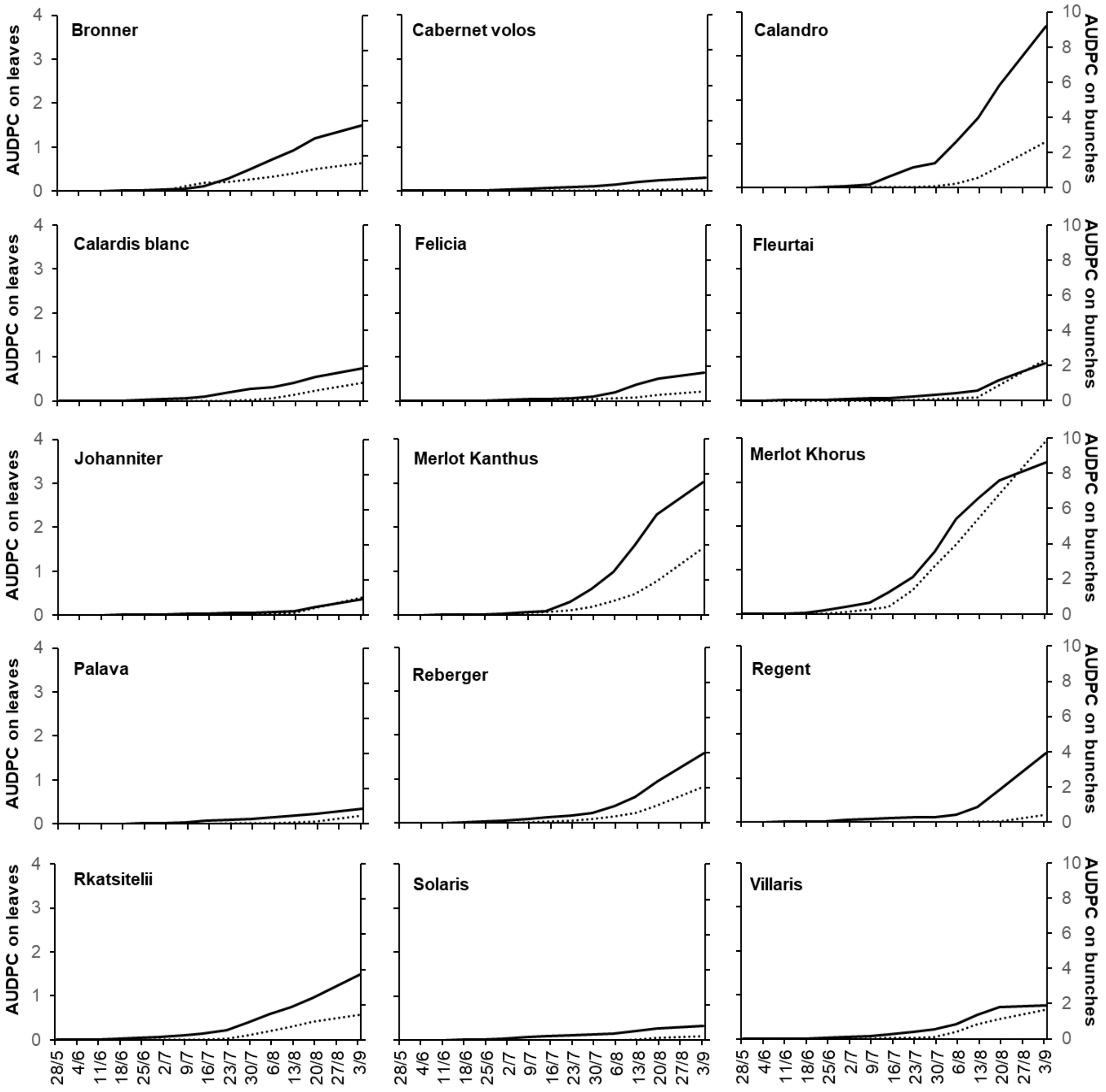

Figure S16. Data for AUDPCs for black rot on leaves (solid line) and bunches (dotted line) recorded on the partially resistant varieties during 2021.
